# Abstract perceptual choice signals during action-linked decisions in the human brain

**DOI:** 10.1371/journal.pbio.3002324

**Published:** 2023-10-10

**Authors:** Florian Sandhaeger, Nina Omejc, Anna-Antonia Pape, Markus Siegel

**Affiliations:** 1 Department of Neural Dynamics and Magnetoencephalography, Hertie Institute for Clinical Brain Research, University of Tübingen, Tübingen, Germany; 2 Centre for Integrative Neuroscience, University of Tübingen, Tübingen, Germany; 3 MEG Center, University of Tübingen, Tübingen, Germany; 4 Graduate Training Centre of Neuroscience, International Max Planck Research School, University of Tübingen, Tübingen, Germany; 5 German Center for Mental Health (DZPG), Tübingen, Germany; Radboud Universiteit Donders Institute for Brain Cognition and Behaviour, NETHERLANDS

## Abstract

Humans can make abstract choices independent of motor actions. However, in laboratory tasks, choices are typically reported with an associated action. Consequentially, knowledge about the neural representation of abstract choices is sparse, and choices are often thought to evolve as motor intentions. Here, we show that in the human brain, perceptual choices are represented in an abstract, motor-independent manner, even when they are directly linked to an action. We measured MEG signals while participants made choices with known or unknown motor response mapping. Using multivariate decoding, we quantified stimulus, perceptual choice, and motor response information with distinct cortical distributions. Choice representations were invariant to whether the response mapping was known during stimulus presentation, and they occupied a distinct representational space from motor signals. As expected from an internal decision variable, they were informed by the stimuli, and their strength predicted decision confidence and accuracy. Our results demonstrate abstract neural choice signals that generalize to action-linked decisions, suggesting a general role of an abstract choice stage in human decision-making.

## Abstract 2

Humans can make abstract choices independent of motor actions, but in lab tasks, choices are typically reported with an associated action. This study shows that the human brain encodes perceptual choices independently of the specific motor actions used to implement them, even if such abstraction is not required by the task context.

## Introduction

Sensory decisions are often linked to an appropriate motor action. This has led to a framework of choices emerging as action intentions [1], supported by numerous studies showing action-specific choice signals in motor and premotor areas of the brain [2–5]. Compelling evidence favors such an intentional framework over the historic idea of decision-making as a sequential process involving several, successive modules. However, a key component of intelligent behavior is the ability to also make abstract choices when a suitable action is not known in advance [6–8]. Any comprehensive account of human decision-making thus has to account for the possibility of abstract choices.

Since most studies use a fixed mapping of perceptual choices (in the following referred to as “choices”) to motor responses, the role of abstraction in sensorimotor decision-making remains elusive. A few notable exceptions, using behavioral tasks with a variable mapping of choices to motor responses, have identified neural representations of abstract choices [7,9–17]. However, empirical results comparing choice signals in action-linked and action-independent situations are sparse. While some recent work found perceptual choice representations to depend on the ability to plan motor actions [5,13] or response modality [18,19], other previous evidence suggests at least partially overlapping representations of perceptual choices with specified or unspecified motor actions [9].

It is therefore unclear whether, and under which conditions, the same neural representations underlying abstract choice in an action-independent context are also present during choices that are linked to actions. If this were not the case, it would suggest that abstract processing is readily bypassed or attenuated when the choice context does not require it. Furthermore, the spatiotemporal dynamics of abstract choice signals are unknown, and it remains unclear whether abstract choice signals constitute an internal decision variable that tracks accumulated evidence. Consequentially, the demonstration of a context-independent, abstract decision variable would be important to confirm predictions of abstraction as an essential stage in perceptual decision-making.

To address this, we investigated human brain activity underlying flexible sensorimotor choices using magnetoencephalography (MEG). The task design and a multivariate analysis framework allowed us to pinpoint abstract neural choice signals in an action-linked as well as in an action-independent context. MEG activity was predictive of participants’ perceptual choices independently of both sensory input and motor behavior. Crucially, a novel metric for the assessment of cross-decoding results enabled us to conclude that abstract choice representations were not only present in both contexts, but indistinguishable between them. Furthermore, choice signals dynamically evolved along the sensorimotor hierarchy and predicted both decision confidence and accuracy, thus exhibiting a hallmark property of an internal decision variable. Our results cast doubt on a purely action-based framework and suggest a general role for abstraction in sensorimotor decision-making.

## Results

### Behavior in a flexible sensorimotor decision-making task

**Fig 1:**
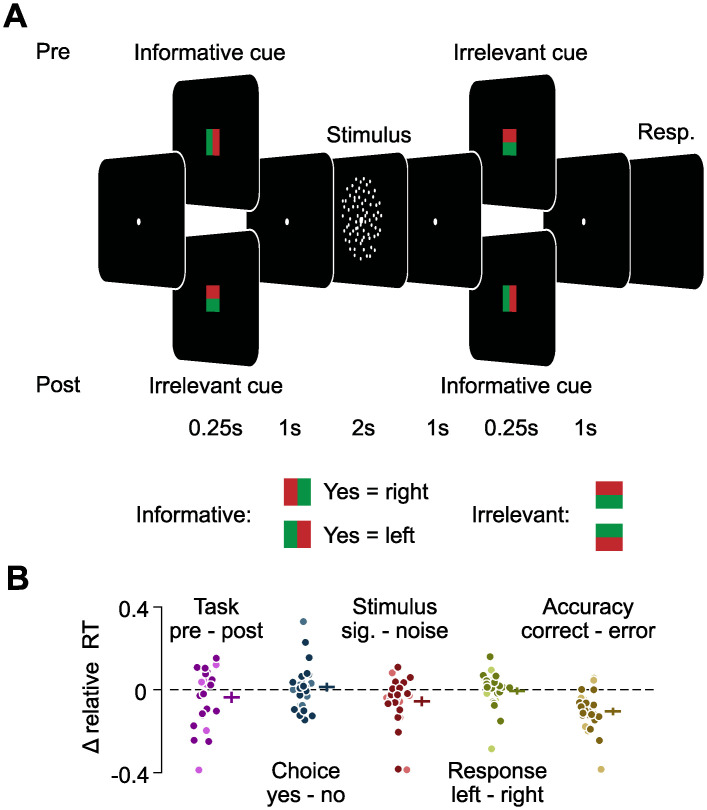
Flexible sensorimotor decision-making task and reaction times. **(A)** In each trial, participants viewed one of 2 random dot stimuli either containing coherent downwards motion (“signal” trials) or containing only random motion (“noise” trials) and reported the presence of coherent motion (“yes” or “no”) with a right- or left-hand button press. Mapping between choice and response was instructed by an informative cue either before (pre-condition, cue 1) or after the stimulus (post-condition, cue 2). Additionally, there was an irrelevant cue offering no additional information either after (pre-condition, cue 2) or before (post-condition, cue 1). Participants additionally used the same button press to indicate their decision confidence, using an inner or an outer button. **(B)** Difference between relative reaction times depending on task, choice, stimulus, response, and accuracy. For each comparison, all other variables were accounted for, and the difference in reaction times was computed after normalizing by the average of both options. Darker and brighter dots indicate participants performing task versions A and B, respectively. Horizontal and vertical bars indicate mean +/− SEM across participants. The data underlying this and all other figures is available at https://osf.io/ucgk4/.

We recorded MEG in 33 human participants, while they performed a sensorimotor decision-making task (Fig 1A, see Methods for subsets of participants used for some analyses). There were 2 slightly different variants of the task, used for different subsets of participants (see Methods). In each trial, we presented one of 2 dynamic random dot stimuli, which either contained coherent downwards motion or not (referred to as “signal” and “noise” trials, respectively), and participants judged the presence of coherent motion. To separate stimulus-related neural signals from choice-related signals, we adapted the coherence level in the signal stimulus for each participant such that they performed near threshold. The presence of both correct and error trials then allowed us to identify neural signals associated with the perceptual choice, independent of the physical stimulus, i.e., neural signals that separated correct signal and incorrect noise trials from incorrect signal and correct noise trials. To disentangle choice- and motor response–related signals, we introduced a flexible mapping between perceptual choices and left- or right-hand button presses that was cued on a trial-by-trial basis. For half of the trials, the choice–response mapping was revealed before stimulus onset (“pre-condition”), such that emerging choices could immediately be linked to the appropriate motor response. For the other half (“post-condition”), we revealed the mapping after stimulus offset, such that participants had to make abstract choices initially, before later selecting their motor response. Participants reported their choices with one of 2 buttons per choice (inner and outer buttons), thereby additionally indicating their confidence. Participants performed equally well on “pre” and “post” trials (74% and 73% correct), neither their sensitivity (d′ = 1.35 and 1.28; *t*_*25*_ = 1.27, *P* = 0.21, two-tailed *t* test) nor criterion (C′ = −0.01 and 0.05; *t*_*25*_ = −1.49, *P* = 0.15) were different between tasks, and neither choice was preferentially associated with a particular motor response (50% “right” responses for both “yes” and “no” choices, *t*_*25*_ = 0.58, *P* = 0.57 and *t*_*25*_ = −0.09, *P* = 0.93, two-tailed *t* test). Furthermore, participants’ performance was not significantly different between coherent and incoherent stimuli (72% and 75% correct responses for coherent and incoherent stimuli, respectively, *t*_*25*_ = −1.41, *P* = 0.17, two-tailed *t* test). In both task conditions, responses had to be withheld until the fixation point disappeared, and while reaction times (0.74 +/− 0.23 s, mean +/− standard deviation over participants) were higher in the post- than the pre-condition (Fig 1B, 0.75 s versus 0.72 s, *F*(1,415) = 7.77, *P* = 0.0056), in noise than in signal trials (*F* = 9.7, *P* = 0.002), and in incorrect than in correct trials (*F* = 34.41, *P* < 10^−8^), they were not significantly different between choices (*F* = 0.81, *P* = 0.37) or responses (*F* = 0.17, *P* = 0.68) (six-way ANOVA including the factors of participant, task condition, stimulus, choice, response, and accuracy).

### Decoding neural representations of stimulus, response, and choice–response mapping

For each task condition separately, we quantified neural information about the stimulus, response, choice–response mapping, and choice using a multivariate analysis approach (cross-validated MANOVA [20,21]; S1 Fig). This method is a generalization of the commonly used cross-validated Mahalanobis distance. cvMANOVA builds on a multivariate general linear model to assess the cross-validated variability contained in the data that is related to a specific variable of interest. While conceptually similar to decoding algorithms, cvMANOVA offers a number of advantages. First, it allows for the simultaneous extraction of information about multiple variables without repeatedly training decoders on each variable separately. Second, this enables the quantification of information related to one variable, while excluding confounds related to any other variable. Third, the resulting measure of the separability of the multivariate activity patterns associated with the variables of interest is continuous, offering a better interpretability and higher sensitivity compared to classifier accuracy. In addition, cross-validation ensures the unbiased estimation of information by using nonoverlapping test and training data sets. Thus, importantly, this analysis isolated neural information about each individual variable, independently of the others. Choice information, for example, was the information contained in the neural data about a participant’s perceptual choice independent of all other variables.

**Fig 2:**
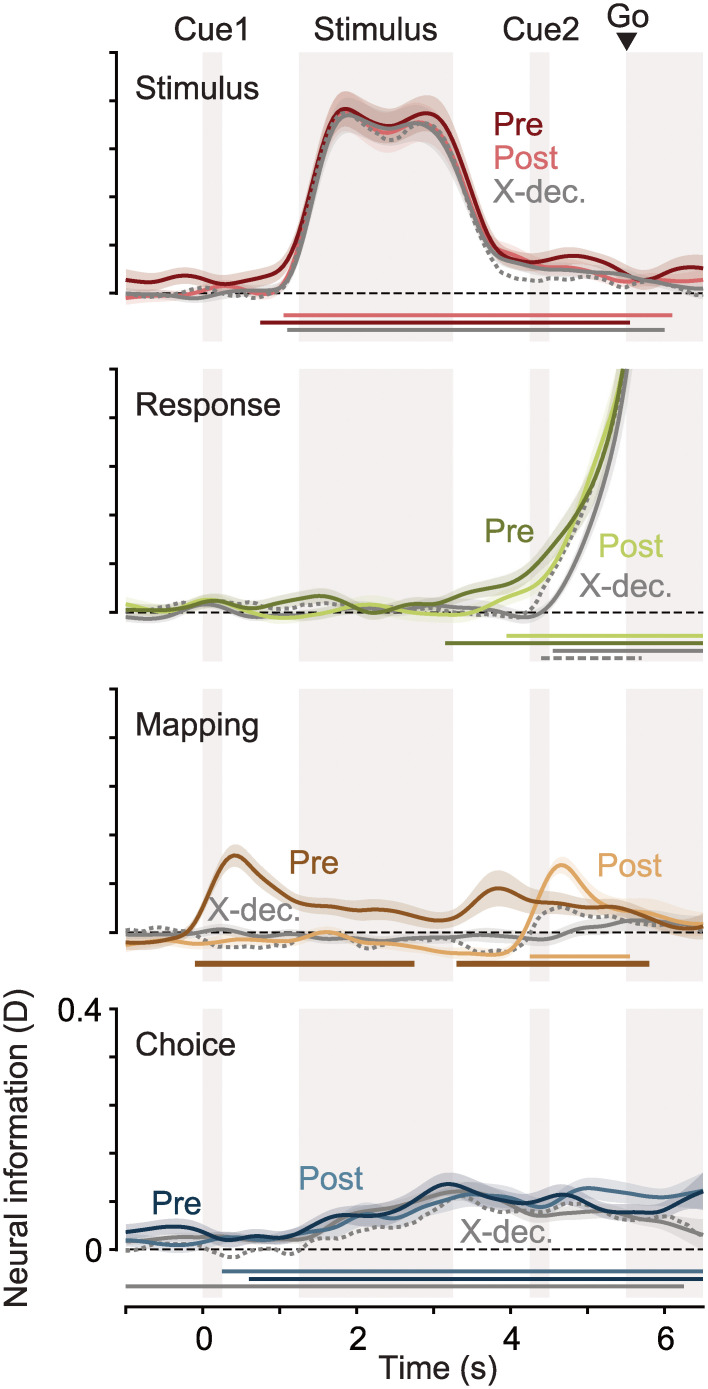
Neural information about the stimulus, response, mapping, and choice. Darker lines indicate information during the pre-condition, brighter lines during the post-condition. Gray lines show the cross-decoding (“X-dec.”) between both conditions, dashed gray lines the cross-decoding expected if representations in both contexts were identical. Horizontal lines denote temporal clusters of significant information (colored lines, *P* < 0.01, cluster permutation, one-tailed, *N* = 26), cross-information (gray, two-tailed) or significantly less cross-information than expected (dashed gray, one-tailed). Coloured lines and shaded regions indicate the mean +/− SEM of information across participants.

We found significant neural information about all task variables in both conditions (*P* < 0.01, cluster permutation statistics, Fig 2). Stimulus information (i.e., the neural pattern distinctness between “signal” and “noise” trials) rose after stimulus onset and remained partially present after stimulus offset. Response information (i.e., right- versus left-hand button presses) built up after stimulus offset; it did so earlier in the pre-condition where the choice–response mapping was already known during stimulus presentation. Motor responses could be predicted more easily, and earlier in the trial, from motor-cortical beta lateralization (S2 Fig; [22]). Choice–response mapping information (i.e., yes/left and no/right versus yes/right and no/left trials) peaked upon presentation of the relevant cue, after the pre-cue in the pre-condition and after the post-cue in the post-condition. Notably, mapping information could in principle be driven by both the visual features of the cue itself and a neural representation of the mapping rule.

### Abstract choice representations generalize between task contexts

Crucially, we also found information about the perceptual choice (i.e., yes versus no choices, Fig 2, bottom, *P* < 0.0001 in both “pre” and “post” conditions, cluster permutation). Even though participants’ choices were related to the presented stimuli and behavioral responses, our analysis framework ensured that choice information could not be explained by neural variability due to either stimuli or responses. Thus, choice information was stimulus and response independent. In both conditions, choices could be predicted before stimulus onset (pre: *P* = 0.003, post: *P* = 0.045; one-tailed *t* tests on time-averaged choice information up to 1.25 s), indicating that they were partly based on purely internal priors.

While, in the “post”-condition, the required motor action was not specified until after the stimulus, choices could be immediately mapped to the appropriate response in the “pre”-condition. Nevertheless, choice information was present in both conditions with a similar magnitude and time course (*P* > 0.05 for all time points before the end of the stimulus, two-tailed *t* test), rising during stimulus presentation and remaining present until the end of the trial. Choice information could not be explained by eye movements (S3 Fig). Thus, choices were represented abstractly in the human brain, regardless of whether they could be directly linked to an action or not.

We employed a cross-decoding approach to assess the extent to which these choice representations were similar between both task conditions. We trained a decoding model on one task condition and tested it on the other. As the information estimated using cvMANOVA is symmetric with respect to the test and training data used, we averaged results from both directions for all cross-decoding analyses. If the multivariate neural patterns distinguishing choices were identical in the “pre”- and “post”-conditions, we would expect the magnitude of the resulting cross-information to be comparable to the information found within the individual conditions. If, on the other hand, choices were represented in orthogonal neural subspaces in both conditions, cross-information should be much lower or negligible.

Cross-decoding of choices was positive throughout the trial (Fig 2, bottom, gray line, *P* < 0.0001, cluster permutation). Furthermore, the magnitude of cross-information was similar to the magnitude of choice information in the “pre”- and “post”-conditions. To quantify this, we derived an estimate of the expected cross-information under the assumption of identical representations in both conditions, i.e., representations relying on the same multivariate pattern and differing only in signal-to-noise ratio between conditions (see Methods). We found that cross-decoded choice information was never significantly lower than expected if representations were identical (*P* > 0.05 for all time points). Thus, abstract choice representations were not only present but were also shared between an action-linked and an action-independent choice context.

All these results were highly similar between both variants of the task, indicating that the slight differences between variants were not relevant for these results (S7 Fig).

### Choice representations dynamically shift from sensory to motor areas

**Fig 3:**
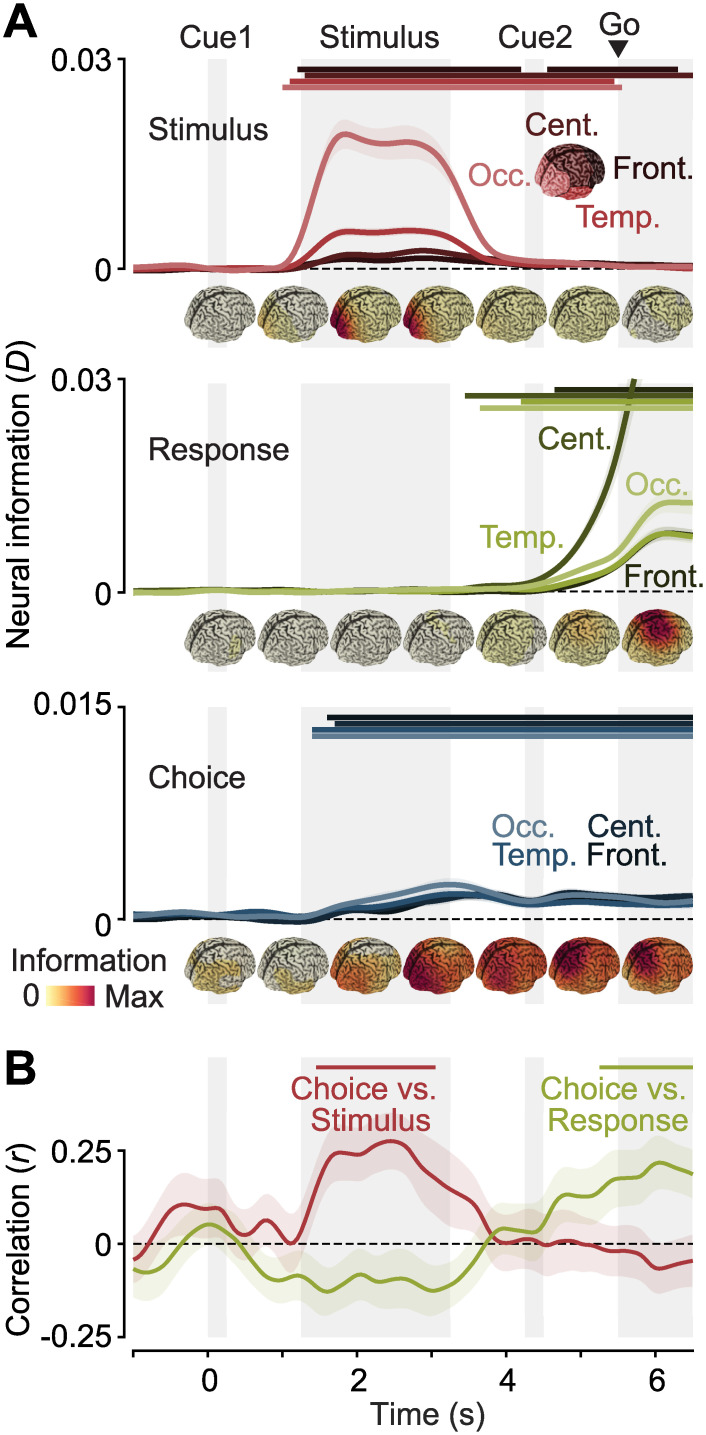
Spatiotemporal dynamics of neural information. **(A)** Time-resolved stimulus (top), response (middle), and choice (bottom) information in 4 groups of sources (in descending order of brightness: occipital, temporal, central, and frontal). Data from both hemispheres were averaged. The cortical distribution of information during different time intervals is shown underneath the time-courses. **(B)** Correlation of the cortical distribution of choice information with the distribution of peak stimulus information (red) and peak response information (yellow). Horizontal lines denote temporal clusters of significant information (A, *P* < 0.05, cluster permutation, one-tailed, *N* = 26) or correlation (B, *P* < 0.05, cluster permutation, one-tailed, *N* = 26). Colored lines and shaded regions indicate the mean +/− SEM of information or correlation across participants.

We further investigated the properties of neural stimulus, choice, and response representations by pooling data from both task conditions. This choice was justified by our finding of shared choice representations and maximized the signal-to-noise ratio for the following analyses. We repeated the decoding analysis in a searchlight fashion across cortex to extract the spatiotemporal evolution of neural information about each variable (Fig 3). During stimulus presentation, stimulus information was strongest in occipital visual cortex, in line with early visual representations of the sensory input. After stimulus offset, information remained at a lower level, uniformly across the brain (Fig 3A, top). Response information increased earliest and most strongly in motor areas (Fig 3A, middle), consistent with preparatory activity related to the upcoming motor response.

The expected cortical distribution and temporal evolution of choice information is less clear. Choices may be represented in visual areas, consistent with findings of choice probabilities in sensory neurons reflecting either the effect of sensory noise on decision formation or high-level feedback onto sensory populations [23–25]. Choice-specific signals may also be present in motor and premotor areas, supporting the planning of potential motor responses [9,18,25,26] or in associative areas specialized for decision formation.

We found that the distribution of choice information changed dynamically over the course of the trial, rising first in occipital areas, before spreading throughout the brain. After the go cue, choice information remained strongest in parietal cortex and central motor areas (Fig 3A, bottom). Given the apparent shift of choice information from occipital areas during stimulus presentation to central areas during the response phase, we quantified the similarity the cortical distribution of choice information exhibited with those of stimulus and response information. We found a significant correlation between the cortical distributions of choice and stimulus information during stimulus presentation, and between choice and response information during the response phase (Fig 3B, stimulus: *P* = 0.0064, response: *P* = 0.0227, cluster permutation). We found similar results when repeating the searchlight analysis independently for pre- and post-condition trials and extracting correlation values for the early stimulus-related and the later response-related cluster. Despite the reduced number of trials, 2 out of 4 correlation values were significant, and all 4 had the same directionality as in the pooled data (stimulus versus choice in pre: *t*_*25*_ = 4.24, *P* = 0.0001, response versus choice in post: *t*_*25*_ = 2.81, *P* = 0.0047, stimulus versus choice in post: *t*_*25*_ = 0.98, *P* = 0.1683, response versus choice in pre: *t*_*25*_ = 1.64, *P* = 0.0569, all one-tailed *t* tests).

### Temporal stability of neural representations

**Fig 4:**
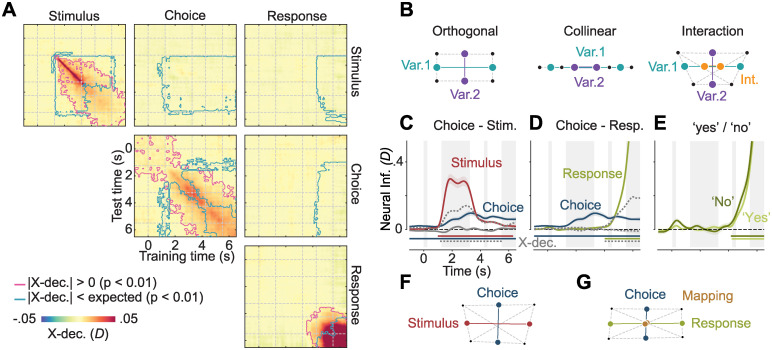
Relationship between stimulus, choice, and response representations. **(A)** Cross-temporal and cross-variable decoding. Colors indicate neural information when trained and tested on any pair of time points and variables. Pink outlines indicate clusters of shared information between time points and variables, i.e., pairs of time points and/or variables during which cross-information is significantly different from 0 (|X-dec.| > 0, cluster permutation, *P* < 0.01, *N* = 26, two-tailed); blue outlines indicate different representations between time points and variables, i.e., pairs of time points and/or variables during which cross-information is significantly smaller than expected for identical representations (|X-dec.| < expected, one-tailed). **(B)** Possible relationships between the representations of 2 variables. Points indicate average activity patterns for different conditions, distances between points the strength of information. Representations may be orthogonal, collinear, or orthogonal but linked with an interaction. **(C** and **D)** Cross-variable decoding between choice and stimulus, and choice and response, respectively. Colored lines show neural information about each variable, gray lines cross-variable information (X-dec.), and dashed gray lines the expected cross-information if both variables were represented identically. Horizontal lines indicate clusters of significant information (colored, *P* < 0.01, one-tailed), or significantly less cross-information than expected (dashed gray, *P* < 0.01, one-tailed). **(E)** Response information for “yes” and “no” choices. Colored lines and shaded regions in panels C, D, and E indicate the mean +/− SEM of information across participants. **(F)** Visualization of the relationship between stimulus and choice representations, based on the cross-decoding values in (C). Stimulus and choice are nearly orthogonal. **(G)** Visualization of the relationship between choice and response representations, including mapping as their interaction, based on (D) and (E). Choice and response representations are nearly orthogonal, and response representations are equally strong for both choices. Thus, there is no systematic relation between the neural patterns encoding choice, stimulus, and response.

The spatial overlap between choice, stimulus, and response information raised the question whether there were shared representations between stimulus and choice during evidence accumulation and between choice and response during motor execution, respectively. We used cross-temporal and cross-variable decoding to test this and evaluated both the temporal dynamics of representations and the relationships between stimulus, choice, and response representations (Fig 4A).

First, we focused on the temporal dynamics of representations. By using data from one time point for training, and from another time point for testing, cross-temporal decoding can reveal time periods of relative stability [27]. Furthermore, it is possible to compute the expected cross-temporal decoding under the assumption that the underlying representation remains perfectly stable over time. Comparing the empirical cross-temporal decoding to this expectation can reveal periods of relative dynamics [28]. Stimulus information was initially highly dynamic, as indicated by high cross-decoding values being concentrated along the diagonal, but stable after stimulus offset, as indicated by significant cross-decoding far from the diagonal (Fig 4A, top left). Given our use of fixed random dot patterns, this was consistent with stimulus information being driven by 2 components: During stimulus presentation, information was likely dominated by moment-to-moment differences in retinal input. After stimulus offset, the global motion content may have contributed more strongly. Choice information was temporally more stable; still, early and late choice representations were distinct (Fig 4A, center), in line with the observed spatial shift from sensory to motor areas.

### Choice representations are distinct from sensory and motor representations

How did the neural representations of different variables relate to each other? The multivariate patterns that encode any 2 variables are either orthogonal, indicating nonoverlapping underlying population subspaces, collinear, indicating indistinguishable circuits underlying both representations, or somewhere in between (Fig 4B; see also S1 Fig for further details). Furthermore, the representation of one variable may differ depending on the value of the other, i.e., the 2 variables may interact. In the present data, stimulus and choice representations may depend on identical underlying circuits. For example, sensory neurons may show the same responses for visually presented as for imagined motion [17,29]. If such neurons constituted stimulus and choice representations, we would expect strong positive cross-information between stimulus and choice. In contrast, if choice and stimulus information were largely driven by distinct populations, this may result in weak cross-information; Our results were compatible with the latter scenario. There was no significant cross-decoding between stimulus and choice (Fig 4A, top center, biggest cluster: *P* = 0.11 and Fig 4C, biggest cluster: *P* = 0.22), and cross-decoding was significantly lower than expected for identical representations (Fig 4A, top center, and Fig 4C, *P* < 0.0001).

Next, we investigated the relationship between choice and response representations. Again, we found only weak cross-information between the 2 variables, indicating that neural choice and response representations did not overlap (Fig 4A, middle right, biggest cluster: *P* = 0.15, and Fig 4D, biggest cluster: from 0.8 to 2.55 s, *P* = 0.038, uncorrected). After selection of a motor response, choices may still have been represented as a modulation of the motor signal, e.g., leading to a relative strengthening of the activity pattern associated with the upcoming motor response for “yes”-choices compared to “no”-choices. We thus assessed the magnitude of response information, separately for each choice. However, we found no difference between both conditions (*P* > 0.05 for all time points), indicating that even during response execution, choices were not represented as a modulation of neural motor activity. (Fig 4E). We further visualized these results geometrically, which well illustrated the near-orthogonality of choice and stimulus, or choice and response signals, respectively (Fig 4F and 4G). In sum, the neural circuit patterns underlying choice information in our MEG data were not significantly shared with those underlying stimulus and response information, even when they were strongest in similar areas.

Abstract choice signals may also be related to, and caused by, sequential choice biases, i.e., preceding choices [25,30,31]. Furthermore, when pooling over the pre- and post-conditions, the higher signal-to-noise ratio revealed robust pre-stimulus choice information (Fig 4A, 4C and 4D), indicating the formation of choices even before stimulus presentation, which may well be related to sequential choice biases. We therefore repeated the analysis including the previous choice as an additional variable and found that choice information after stimulus onset could not be explained by the previous choice (S4A Fig). Including the previous motor response instead of previous choice showed a sustained representation of past motor actions [22]. However, this had an even weaker effect on choice information. Thus, neuronal choice signals did not merely reflect the previous choice or motor response.

**Fig 5:**
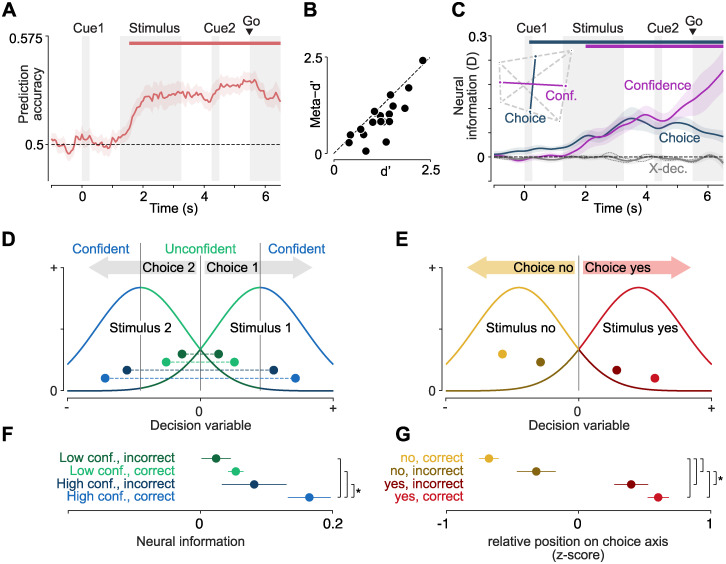
Choice representations behave like a decision variable. **(A)** Prediction of stimulus class from the sign of single trial choice information. Mean +/− SEM across participants. **(B)** Behavioral sensitivity (d′) and meta-cognitive sensitivity (meta-d′). **(C)** Neural information about choice and confidence, as well as cross-variable decoding between the two. Dotted lines indicate cross-variable decoding using only participants performing task versions A and B, respectively. Horizontal lines denote temporal clusters of significant information (colored lines, *P* < 0.01, cluster permutation, one-tailed, *N* = 19. Colored lines and shaded regions indicate the mean +/− SEM of information across participants. The inset shows a visualization of the relationship between choice and confidence representations, based on the cross-decoding values. Choice and confidence are nearly orthogonal. **(D)** The relationship between decision variable, confidence, and accuracy as predicted by signal detection theory. For each of the 2 stimuli, the distribution of values of the decision variable is centered on the respective side of the decision boundary at 0. When the absolute distance to the decision boundary is larger, the observer is more confident in their choice. Correct and incorrect, confident and unconfident trials are color coded as in (E). **(E)** Time-averaged choice information (1.25 to 4 s) in trials split by confidence and accuracy. Stars denote significance (*P* < 0.05, one-tailed *t* test, *N* = 19). **(F)** The relationship between decision variable, accuracy, and choice as predicted by signal detection theory. For both yes- and no-choices, the decision variable has a higher absolute magnitude in correct trials. Correct and incorrect, yes and no trials are color coded as in (G). **(G)** Time-averaged (1.25 to 4 s), normalized placement on the choice axis of trials split by choice and accuracy. Stars denote significance (*P* < 0.05, one-tailed *t* test, *N* = 19).

While our analysis already excluded the possibility that choice information was driven by overall differences between stimuli, it could theoretically still be explained by a difference between correct and error trials for one of the 2 stimulus classes. To eliminate this possibility, we trained the choice decoding model on all trials and evaluated it separately on correct and incorrect trials. As choice information was present in both cases, and had the same sign, it could not be explained by choice accuracy (Fig 5G). In sum, abstract choice information did not result from the representation of either previous choices or accuracy as potentially confounding variables.

### Choice signals predict stimuli

The above results suggested that choice signals reflected an abstract decision stage that was distinct from both early sensory and motor representations. Nonetheless, behavioral choices were based on the presented stimuli and therefore strongly correlated with them. We thus asked whether this relationship was reflected in the neural choice signals, or whether they were stimulus independent and therefore purely internally driven. To do so, we computed single-trial estimates of the choice signal by projecting each trial’s data onto the choice axis defined by our multivariate analysis. We then assessed whether the sign of the single-trial choice signals predicted the stimulus. If the choice signal were purely internally driven, we would expect this stimulus predictability to be at chance even in the presence of significant choice information. Conversely, an influence of the stimulus on the choice signal would lead to above-chance stimulus predictability. Indeed, after stimulus onset, the predictability of the stimulus increased until it reached a stable level for the remainder of the trial (Fig 5A, *P* < 0.0001, cluster permutation). As expected, there was no significant stimulus predictability before stimulus onset, even though there was a small amount of choice information (Fig 4C).

### The strength of choice signals predicts decision confidence and accuracy

Our participants also reported their confidence in each trial’s perceptual choice, providing us with further leverage to unravel the nature of the choice signals we found. Specifically, this allowed us to test whether the choice-predictive signal merely correlated with choices, or whether its relation to accuracy and confidence exhibited additional key properties expected of a decision variable integrating evidence towards a choice.

First, we behaviorally assessed the relationship between participants’ choices and confidence ratings. Participants were more confident in yes- than in no-choices (average proportion of high confidence reports: 0.54 versus 0.47, *P* = 0.034, two-tailed *t* test) and in trials with signal than in those with noise stimuli (0.55 versus 0.46, *P* = 1.4 × 10^−4^). In addition, and critically, they reported high confidence more often in correct trials than in incorrect trials (average proportion of high confidence reports: 0.56 versus 0.35, *P* = 3 × 10^−7^, two-tailed *t* test). We quantified this relationship using the meta-d′ measure of metacognitive sensitivity [32] (Fig 5B). As expected, meta-d′ was positive (0.93 +/− 0.55, mean +/− standard deviation over participants, *t*_*18*_ = 7.4, *P* = 7.5 × 10^−7^, two-tailed *t* test), correlated with d′ (r_*17*_ = 0.82, *P* = 1.5 × 10^−5^, Pearson correlation), but tended to be smaller than d′ (1.23 +/− 0.49, mean +/− standard deviation over participants, *t*_*18*_ = −4.1, *P* = 7 × 10^−4^, two-tailed *t* test). This showed that participants veridically reported their confidence and suggested that their confidence judgements were largely, but not perfectly based on the same sensory evidence as their choices [33–36]. These results also held when we separately assessed them in the pre- and post-conditions, and neither d′ (*t*_*18*_ = 1.2, *P* = 0.24) nor meta-d′ (*t*_*18*_ = 0.2, *P* = 0.83) were significantly different between conditions.

Secondly, we asked whether confidence was represented in the neural data. We thus repeated our decoding analysis, now adding decision confidence as an additional variable. There was significant neural information about confidence, starting around stimulus presentation and continually rising until the end of the trial (*P* < 0.01, cluster permutation statistics, one-tailed, Fig 5C, pink line). Furthermore, also choice information remained significant and apparently unchanged when adding confidence as an additional variable (*P* < 0.01, cluster permutation statistics, one-tailed, Fig 5C, blue line). Given that behavioral confidence slightly correlated with the participants’ choices, choice information could in principle be confounded by signals associated with the confidence reports. However, simultaneously including both variables in the analysis effectively isolated their contributions. Thus, the remaining choice information was independent of the confidence reports. To further assess whether residual variability of confidence within reports could account for the choice signal, we employed cross-variable decoding between choice and confidence. We found no overlap between both variables’ neural representations, indicating that this was not the case (*P* > 0.05 for all time points, two-tailed, Fig 5C, gray line and inset). This result was the same in both task versions (*P* > 0.05 for all time points, two-tailed, Fig 5C, gray dotted lines), despite a consistent confidence–response mapping in task version A and a counterbalanced confidence–response mapping in task version B. Thus, choice information in our data was not confounded by either the confidence reports or residual variability of confidence.

Next, we directly investigated the relation of neural choice signals to decision confidence and accuracy. In signal detection theory and in related accumulator models of decision-making, an internal decision variable tracks the integrated evidence for a given choice. Importantly, such a decision variable enables the computation of choice–confidence, as the absolute distance to the decision boundary [37–40]. Consequentially, the absolute value of the decision variable should be larger during high-confidence trials than during low-confidence trials (Fig 5D, blue versus green), and, importantly independent of confidence, higher during correct than during error trials (Fig 5D, bright versus dark colors).

To establish whether the choice signals found here could reflect an internal decision variable, we trained the decoding model separately on confident and unconfident trials and tested it separately on confident correct, unconfident correct, confident error, and unconfident error trials. We hypothesized that, if choice information constituted an internal decision variable reflecting the same subjective evidence used to inform confidence judgements, it would be strongest in correct trials when confidence was high, and progressively weaker in confident error trials, unconfident correct trials, and unconfident error trials.

Indeed, we found that the strength of choice representations descriptively followed this pattern predicted by signal detection theory (Fig 5F: correct/high confidence larger than incorrect/high confidence, correct/low confidence, and incorrect/low confidence; *P* = 0.013, *P* = 0.002, *P* = 0.001; high confidence larger than low confidence and correct larger than incorrect; *P* = 0.027, *P* = 0.025). Importantly, participants’ accuracy and confidence were assessed as separate factors. Thus, the relationship between choice and confidence could not simply be explained by accuracy or vice versa. We additionally performed this analysis separately for the pre-cue and post-cue task conditions, after excluding the factor of response from our model in order to retain a sufficient number of trials per condition. There was a similar pattern in both tasks (post: correct/high confidence larger than incorrect/high confidence, correct/low confidence, and incorrect/low confidence; *P* = 0.035, *P* = 0.008, *P* = 0.005. pre: correct/high confidence larger than incorrect/high confidence, correct/low confidence, and incorrect/low confidence; *P* = 0.007, *P* = 0.002, *P* = 0.023). In contrast, there was no clear relationship between choice confidence or accuracy and the strength of stimulus or motor representations (S5 Fig).

Finally, we investigated the relative placement of correct and incorrect trials of both choices on the neural choice axis. As the stimulus design used was inherently asymmetric (signal versus noise stimuli), a similar asymmetry may be expected for the neural representations of yes- and no-choices, opening the door for potential, choice-unrelated confounds. For example, the timing of choice commitment may be different for yes- and no-choices, differentially affecting the neural signal. While our fixed-time design did not provide access to commitment times, there is one previous study investigating reaction times in a similar forced-response detection task. In that study, the authors found responses to be slower for no- than for yes-choices, and highly similar between correct and incorrect no-choices [41]. This is consistent with no-choices occurring when the internal decision variable does not hit a bound until the response is made. This leads to a critical prediction for the present data. If neural choice signals reflected the time of choice commitment rather than the decision variable itself, they should exhibit a similar pattern with a difference between yes- and no-choices but similar signals for correct and incorrect no-choices. To test this, we trained a decoding model on all choices and tested it separately on, first, correct yes versus correct no-choices; second, incorrect yes versus incorrect no-choices; and third, correct yes versus incorrect no-choices. The resulting distances allowed us to estimate the relative placement of correct and incorrect, yes and no choices on the choice axis. As expected from a neural decision variable, these trial types were well ordered, with correct no-choices being followed by incorrect no-, incorrect yes-, and correct yes-choices (Fig 5E and 5G, *P* < 0.05 for all pairwise comparisons apart from correct yes versus incorrect yes with *P* = 0.12, one-tailed *t* tests). In contrast, this ordering does not fit well with a timing confound arising from a potential asymmetry between yes- and no-choices.

In sum, our behavioral results pointed to the existence of an internal decision variable, which informed both choices and confidence ratings. Furthermore, the strength of the choice-predictive neural signal varied with confidence and accuracy, precisely following a pattern predicted from signal detection theory. Thus, neural choice information measured in MEG did not only predict abstract perceptual choices but appeared to reflect the underlying internal decision variable.

## Discussion

Studies of the neural basis of sensorimotor decision-making have often neglected abstract, motor-independent choices [42]. This is rooted in the fact that many real-world choices appear to be choices between motor actions [1] and in the difficulty of accessing signals representing purely abstract choices. On the one hand, in animal studies, which provide the majority of evidence in support of neural circuits selective for specific choice options, behavioral tasks that disentangle choices from motor responses are very challenging. Noninvasive human studies, on the other hand, struggle to read out choice contents and thus mostly provide indirect evidence for choice-related neural activity. Consequently, studies comparing the representation of choices in abstract and action-linked contexts are rare. A small number of notable exceptions have provided intriguing results [5,9,13] but not established a unified account of the role and extent of abstract choice signals.

By combining noninvasive MEG in humans with an advanced multivariate analysis framework, we robustly read out abstract choice contents from whole-brain neural activity. In accordance with current theories [39,40], abstract choice representations predicted decision confidence and accuracy. This indicates that this neural signal did not merely correlate with categorical choice but reflected the underlying decision variable. In sum, our findings point to an important role of abstraction in decision-making, even in a simple task involving a known sensorimotor mapping.

Abstract choices were represented in brain activity not only when decisions had to be made abstractly but also when the sensorimotor mapping was known in advance. Importantly, our cross-decoding analysis showed that choice representations in both task contexts were indistinguishable from one another. While the fundamental limits of MEG spatial resolution and sensitivity prevent the conclusion that the underlying circuit representations are identical, this striking similarity requires any potentially remaining differences between conditions to be small and of a type that MEG is blind to.

Our finding of abstract choice representations generalizing between contexts in which actions can be planned and those in which they cannot is in line with behavioral evidence suggesting analogous mechanisms underlying decision-making in action-linked and action-independent contexts [43]. Moreover, recordings in macaque lateral intraparietal area (LIP) have found the choice selectivity of neurons to be similar, regardless of whether a motor action was specified or not [9]. Our results extend this finding to the whole-brain level, indicating that the dominant sources of choice-selective signals generalize between contexts. Intriguingly, a recent study found representations of the decision variable in area LIP that were not tightly linked to the population’s oculomotor selectivity but varied in a task-dependent manner [44]. These task-dependent representations are compatible with abstract, motor-independent choice representations computed in LIP or elsewhere [45] as reported here. Furthermore, our findings accord well with research implicating a centro-parietal positivity (CPP) as an electrophysiological marker of evidence accumulation [46,47]. The CPP exhibits several properties of a domain-general, abstract-neural decision variable; however, while it gradually builds up with the absolute amount of evidence, it has not been shown to carry information about the choice itself [48]. Thus, the CPP, as an unsigned marker of integration, and the specific choice signals found in the present study may reflect different aspects of the same underlying process.

This—as well as any other—decision-making study lives off the fact that sometimes participants make different choices for identical stimuli. How does this variability arise? In principle, 2 broad, and not mutually exclusive, classes of explanations exist. First, it could be bottom-up driven, with sensory noise having a causal effect on choices. This sensory noise could be internal, arising from the inherent variability in neural responses for identical stimuli, or due to uncontrolled external variability, such as small differences in the stimulus itself. Second, it could be top-down driven, with internal factors such as expectations, biases, or beliefs or simply nonsensory noise pushing choices one or the other way. Several of our results consistently suggest that the demonstrated choice signals are positioned at an intermediate stage between these extremes. First, if the choice signals directly reflected sensory noise, we would expect this noise to inhabit the same neural subspace as the stimulus signals themselves—in other words, there should be strong cross-information between stimulus and choice. In contrast to this, our results are better compatible with choice signals reflecting integrated sensory noise represented distinctly. For example, one may expect to find instantaneous sensory noise represented in the middle temporal visual area (MT), but integrated sensory information, and therefore integrated noise as well, represented in area LIP. Notably, such an integration stage would still be expected to be modulated by the stimulus, which may lead to stimulus-choice cross-information, but only subtly so. In our data, this effect did not result in significant cross-information but was nonetheless apparent in the hypothesis-driven finding of stronger choice signals in correct than in error trials. Second, we found that signed choice information predicted the stimulus—despite near-orthogonality of the representations of both variables. This suggests that choice information was indeed reflective of a stage separate from but influenced by the early sensory representation. This prediction increased during stimulus presentation and then remained stable, similar to the choice information time course itself, and consistent with the time course expected from temporal integration. In contrast, choice signals at an instantaneous, early sensory nonintegration stage would also predict the stimulus, but predictions should be at the same level throughout the stimulus presentation interval, and subsequently taper off. Third, we found small amounts of choice information before stimulus presentation. As these could not have arisen due to the stimulus, they must reflect intraneous factors. In conclusion, the most parsimonious explanation for our data is an intermediate choice stage that reflects both accumulated sensory evidence and top-down contributions, akin to an internal decision variable. Importantly, these considerations allow us inferences about the nature of the measured signal, regardless of its exact anatomical origin. While reflecting an intermediate, abstract stage, the choice signal may well be fed back or forward to sensory or motor populations [24,25,49], respectively, and contribute to MEG decodability in these areas.

The stimuli used in the present study, and therefore the corresponding choices, were inherently asymmetric. One may ask if this asymmetry could underlie the decodablity of choices: An unobserved, confounding variable correlated with choice may result in the seeming readout of choice information. To address this, we ruled out potential confounds associated with stimulus asymmetry. First, our finding of a significant difference of the choice signal between correct and incorrect no-choices is incompatible with a timing-related confound due to this asymmetry [41]. Second, could there be a default no-choice encoded at the beginning of the trial, which may then be modified upon the presentation of the stimulus? The ability to decode choices before stimulus onset suggests the existence of a pre-stimulus prior. However, this prior needs to be variable at least in magnitude, if not in sign, to push the choice either way and thus lead to decodable information. Furthermore, participants performed equally well for coherent and incoherent stimuli, indicating that they did not exhibit a strong bias due to the stimulus asymmetry or any potential default behavior. Third, the slight correlation between behavioral confidence and the participants’ choices could not account for our results because there was residual choice information within each confidence level, and the neural representations of choice and confidence were orthogonal and followed distinct time courses. Even more generally, any confounding variable would have to exhibit the properties of the choice signal demonstrated here: small, but existing pre-stimulus differences between choices, a modulation by confidence and accuracy even within no-trials, and a trial-by-trial predictability of the stimulus. In sum, we ruled out potential asymmetry-related confounds and generally found no indication that the asymmetric task-design could confound our results.

The cortical distribution of abstract choice signals may be modulated by response modality. Recent work using fMRI suggested that, for vibrotactile comparisons, abstract choice representations are present in nonoverlapping, modality-specific cortical areas [19,50]. On the other hand, direct neuronal recordings have shown the representation of recognition and categorization choices in medial frontal cortex to generalize between manual and saccadic responses [16]. The accessibility of such modality-independent representations of choice likely depends on the specific behavioral task and type of neural measurement. Research combining multiple measurement scales [51–53] should help resolving this. Our results only have indirect implications for the modality dependence of choice signals because participants eventually always reported their choice using a button press. Nonetheless, the broad availability of choice representations across the brain, in conjunction with the shift of the information peak from visual sensory to motor areas is consistent with the coexistence of modality-independent and modality-specific components.

Perceptual decisions involve a complex interaction of feedforward and feedback processes throughout the brain [25,49,54]. Here, we found that the spatial peak of abstract choice information shifted throughout the trial, reflecting the currently relevant stage [25,55]. This does not necessitate that choices originate in sensory cortex and are later relayed to motor cortex; in fact, choices may be computed elsewhere but be preferentially accessible in currently engaged areas. The global availability of choice information is in line with either a distributed computation that involves recurrent interactions, or a global broadcast of choice signals [24,25], for example, through feature-attentional mechanisms [49]. Further studies including invasive and manipulative approaches are required to pinpoint where and by which mechanisms abstract choices are computed.

A growing body of evidence has related the formation of action-linked sensorimotor decisions to activity in motor and premotor areas [2,3,5,26,42,54]. Our findings are well compatible with these results: The presence of an abstract choice stage does not preclude the simultaneous planning and competition of multiple response options or a general unspecific response preparation [26,56]. Indeed, we found fluctuations in motor cortical beta band activity to predict upcoming motor responses, independently of the perceptual choice [22,57]. These response-predictive beta band signals ramped up upon stimulus presentation in the “pre”-condition, as expected due to the earlier availability of the choice–response mapping. Notably, this ramp-up happened earlier than the appearance of response information in the broadband electrophysiological signals, underpinning the well-known role of beta band activity as a specific marker of motor preparation [47]. This indicates that in the case of a known physiological marker such as the beta-band lateralization, a targeted analysis can be more sensitive than the uninformed whole-brain decoding strategy employed throughout this study. Taken together, these findings support a multilevel model of decision-making involving simultaneous evaluation of abstract choices as well as motor actions [58]. The relevance of an abstract choice level may be understood in light of phenomena like perceptual priors [59,60], sequential choice biases [22,31], or value computations associated with the choices themselves [61], which all require and act on abstract choice representations. The primate brain, which is able to assess abstract options and treat decision-making problems as arbitrary categorization [15,62], may do so even when not strictly necessary. Importantly, this can still be reconciled with an intentional framework of decision-making, if intentions are not only about actions, but also rules, or activations of neural circuits in general [39,58].

We conclude that an abstract choice stage may be universally present in human perceptual decision-making, enabling the evaluation of motor-independent choice options even during action-linked decisions.

## Methods

### Ethics statement

Participants provided written informed consent prior to the start of the experiment. The study was conducted in accordance with the Declaration of Helsinki and was approved by the ethical committee of the Medical Faculty and University Hospital of the University of Tübingen (approval number 419/2011 B02).

### Participants

A total of 33 healthy, right-handed human volunteers (18 female; mean age: 28 y; 3 y SD) participated in this study and received monetary reward. All participants had normal or corrected-to-normal vision.

### Behavioral task and stimuli

Participants performed a flexible sensorimotor decision-making task. In each trial, they had to decide whether a random dot kinematogram contained coherent downwards motion or not and reported their choice with a left- or right-hand button press. Crucially, the mapping between response hand and choice varied on a trial-by-trial basis. Moreover, the mapping was revealed either before (pre-condition) or after (post-condition) the stimulus. Additionally, an irrelevant cue was presented after (pre-condition) or before (post-condition) the stimulus.

Participants started a trial by acquiring fixation on a fixation spot. After a fixation period, the first cue appeared for 250 ms, followed by a delay of 1,000 ms, the presentation of the random dot stimulus for 2,000 ms, another 1,000-ms delay, and the second cue for 250 ms. A third 1,000-ms delay was followed by a 33-ms dimming of the fixation spot, which served as the go-cue for the participant’s response. The response consisted in a button press using the left or right index finger, according to the choice and the choice–response mapping. Participants chose one of 2 buttons on either side to indicate whether they were confident in their choice or not. Participants received a 100-ms visual feedback (centrally presented circle, 2.1 degree diameter, red for incorrect or green for correct), 250 ms after their response.

The random dot stimuli consisted of 1,500 white dots with a diameter of 0.12 degrees, presented in an 8.5 degree diameter circular aperture on a black background. Dots moved at a speed of 10 degrees per second. For each participant, we used only 2 stimuli, each presented in half of the trials: First, a target stimulus, in which, on each frame, a fraction of dots moved coherently downwards, whereas the rest moved in random directions. Second, a noise stimulus, in which all dots moved in random directions. In a separate session before the MEG recordings, the motion coherence of target stimuli was titrated to each participant’s individual perceptual threshold using a staircase procedure with 280 trials. Motion coherence was adaptively lowered by one level after each correct choice and increased by 2 levels after each incorrect choice. To determine the coherence threshold, a Weibull function was fit to the resulting data, excluding the first 50 trials. Choice–response cues and irrelevant cues all had the same luminance and size (0.85 degree diameter).

Each participant took part in 2 recording runs of one of 2 task versions, which differed in the details of the choice–response cue as well as the confidence report. Participants 1 to 20 performed version A: Here, the choice–response cue consisted of a centrally presented red or green square (yes = right hand: green; yes = left hand: red), whereas the irrelevant cue was a blue square. The outer button always indicated a confident, the inner one an unconfident choice. In this version, the fixation baseline at the beginning of each trial lasted 1,500 ms. Each recording run consisted of 400 randomly ordered trials, of which 120 were pre-cue trials, 120 post-cue trials, and 160 belonged to one of 2 control conditions not reported here. Participants 21 to 33 performed version B: Here, the choice–response cue consisted of 2 vertical rectangles (yes = right hand: left rectangle mint, right rectangle pink; yes = left hand: left rectangle pink, right rectangle mint) forming a square, whereas the irrelevant cue consisted of 2 horizontal rectangles (upper: pink, lower: mint). The confidence mapping (inner or outer button for confident/unconfident responses) was changed in each recording run. Here, the fixation baseline was 1,000 ms. Each run consisted of 400 randomly ordered trials, 200 of which were pre-cue and 200 post-cue trials. The changes in version B were designed to minimize sensory and motor confounds in a separate analysis of task- and confidence-related effects (not reported here). The data from version A were previously used in another publication [22].

To ensure that participants were performing both task conditions well, we computed overall accuracy as the percentage of correct trials. We used a two-tailed paired *t* test to test whether accuracy was different between task conditions. To make sure participants did not systematically associate one of the motor responses with one of the choices, we computed the percentage of “right” button presses for “yes” and “no” choices separately and compared both against 50% using two-tailed *t* tests.

### Setup and recording

We recorded MEG (Omega 2000, CTF Systems, Port Coquitlam, Canada) with 275 channels at a sampling rate of 2,343.75 Hz in a magnetically shielded chamber. Participants sat upright in a dark room, while stimuli were projected onto a screen at a viewing distance of 55 cm using an LCD projector (Sanyo PLC-XP41, Moriguchi, Japan) at 60 Hz refresh rate. Stimuli were constructed offline and presented using the Presentation software (NeuroBehavioral Systems, Albany, CA, USA). To ensure continuous fixation, we recorded eye movements using an Eyelink 1000 system (SR Research, Ottawa, Ontario, Canada).

### Preprocessing

We used time-domain data for the decoding analyses. Thus, we low-pass filtered MEG and eye-tracking data at 10 Hz (two-pass forward-reverse Butterworth filter, order 4) and down-sampled to 20 Hz to maximize SNR by reducing the impact of high-frequency noise, to focus our analysis on slow cortical potentials that may be linked to the gradual build-up of a decision variable, and to avoid the necessity for any effects to be precisely temporally aligned across trials. Trials containing eye blinks were rejected. We chose not to apply a high-pass filter in order to avoid filter artefacts [63]. At the same time, we could not use a baseline correction as choice effects could plausibly be driven by previous trials. We thus used robust detrending [64] to remove polynomial trends from the MEG data, but not the eye tracking data, in a piecewise fashion (600-s pieces, removal of linear trend followed by 10th order polynomial). Data of 3 participants was rejected due to metal artifacts.

### Source reconstruction

For source reconstruction based on each participant’s individual anatomy, we recorded structural T1-weighted MRIs (echo time (TE) = 2.18 ms, repetition time (TR) = 2.3 ms, longitudinal relaxation time (T1) = 1.1 ms, flip angle = 9°, 192 slices, voxel size 1 × 1 × 1 mm3) with a Siemens 3T Tim Trio scanner and a 32 channel Head Coil. We generated single-shell head models [65] and estimated three-dimensional (x, y, and z-direction) MEG source activity at 457 equally spaced locations 7 mm beneath the skull, using linear spatial filtering [66]. We retained, for each source, activity in all 3 directions and concatenated the data of the 2 separate recording runs per participant. For all subsequent analyses, we reduced the dimensionality of this 1,371-dimensional source space: For all whole-head decoding analyses, we performed principal component analysis, retaining the 75 components with the largest variance across all combinations of task variables. For searchlight analyses, we used each of the 457 sources’ immediate neighbors, including all 3 dipole directions.

### Task variables and cross-validation scheme

The experimental design resulted in a number of variables of which each trial instantiated a combination. For each trial, we defined the task (pre- or post-cue), stimulus (target or noise), response (left- or right-hand button press), mapping (target = left or target = right), choice (yes/target or no/noise), accuracy (correct or incorrect), and confidence (high or low). Not all of these variables were independent of each other: For a given stimulus and choice, accuracy is fixed; and for a given choice and mapping, response is fixed. Thus, 5 independent variables giving rise to 32 conditions remained (S1A Fig). While those variables under experimental control (task, stimulus, mapping) were fully balanced, those dependent on the participants’ behavior (choice, response, confidence, accuracy) were not, leading to a nonuniform sampling of conditions (S1A Fig). To ensure an accurate estimation of neural information about each variable, independent of the others, we implemented an n-fold cross-validation scheme, where n was the lowest trial count per condition. Thus, for each cross-validation fold, both training and test data contained trials of all conditions. In order to decrease the dependence of our results on a particular random partition into folds, we repeated each analysis 10 times, with different random seeds. All results were averaged across these random seeds before further processing.

Due to the variability in behavioral responses, as well as the rejection of trials containing eye blink artefacts, we did not retain the same amount of trials from each condition for all participants. However, to accurately estimate neural information, we needed to ensure that, first, there were trials of each condition, and second, the total number of trials was large enough in comparison to the dimensionality of the data to enable an unbiased estimate [20]. Specifically, each analysis requires at least N + K + 1 trials, where N is the number of channels and K is the number of independent variables in the model. For our main analyses (Figs 2, 3 and 4, S2 and S3 Figs), including task, stimulus, choice, response, mapping, and accuracy as variables, data from 26 participants had sufficient trials. When additionally including confidence as a variable, but neglecting the task condition (Fig 5 and S5 Fig), we retained 19 participants. To assess the effect of confidence separately for both task conditions, we used all variables apart from response, leading again to 19 usable participants. To assess the effect of the previous choice in relation to the current choice (S4A Fig), we neglected the task condition as well as confidence and included stimulus, choice, response, mapping, accuracy, and previous choice. This left us with data from 23 participants. To assess the effect of the previous motor response in relation to the current choice (S4B Fig), we neglected the task condition as well as confidence and included stimulus, choice, response, mapping, accuracy, and previous response. This left us with data from 25 participants. Importantly, when restricting all analyses to the core subset of 19 participants for which every analysis was possible, our main results were virtually identical (S6 Fig). For all decoding analyses, we combined source level data from both recording runs per participant. Using source-level data allowed us to reduce between-run variance and reduce nonneural variability. To do so, we normalized the data per channel, time point, and run over trials and then concatenated data of both runs.

### Cross-validated MANOVA

We used cross-validated MANOVA [20,21] to estimate the amount of information in multivariate MEG data about the task variables of interest. CVMANOVA estimates the variability explained by the task variables in relation to unexplained noise variability. Here, we reimplemented cvMANOVA for time-resolved data, adding the capability of cross-decoding by training and testing the model on different time points, variables, or levels of any variable. To this end, we first estimated a baseline noise covariance matrix, using trials from all unique conditions. We then “trained” the model by estimating contrasts of beta weights of each unique condition in a cross-validation fold’s training set and “tested” it by estimating contrasts of beta weights in the fold’s test set. An estimate of true pattern distinctness was computed as the dot product of these contrasts, normalized by the noise covariance:

$$D=trace\left(\frac{1}{n}B_{train}^{\prime }C_{train}C_{train}^{-1}X_{test}^{\prime }X_{test}C_{train}C_{test}^{-1}B_{test}\Sigma^{-1}\right)$$

where X_test_ is the design matrix indicating the unique condition of each trial in the test set, C_train_ is the contrast vector the model is trained on, C_test_ the test contrast vector and Σ^−1^ the inverted noise covariance matrix. B_train_ and B_test_ contained the regression parameters of a multivariate general linear model

$$B_{train}=X_{train}^{-1}Y_{train}$$

$$B_{test}=X_{test}^{-1}Y_{test}$$

where Y_train_ and Y_test_ are the training and test data sets. The inverted noise covariance matrix Σ^−1^ was estimated using data from a baseline time point (−0.5 s with respect to the onset of the first cue):

$$B_{train_{bl}}=X_{train}^{-1}Y_{train_{bl}}$$

$$\Xi =Y_{train_{bl}}-X_{train}B_{train_{bl}}$$

$$\Sigma^{-1}=\left(fE-p-1\right)∙{\left(\Xi^{\prime }\Xi \;\right)}^{-1}$$

with fE being the degrees of freedom and p the number of sources used. Ξ was regularized towards the unity matrix using a regularization parameter of 0.05.

Because the design matrix and contrast vector include all unique conditions, i.e., all combinations of variable levels (S1 Fig), cvMANOVA independently quantifies information about each variable of interest, while not being confounded by information about the other, potentially correlated variables. In other words, cvMANOVA quantifies the pattern distinctness explained by each variable after discounting the patterns explained by all other variables included in the model. Importantly, cvMANOVA effectively controls imbalances in the distribution of trials over conditions without explicit stratification and the resulting loss of data.

While cvMANOVA technically constitutes an encoding framework—modelling data variability due to experimental variables—it shares many similarities with commonly used multivariate decoding methods [67]. Notably, cvMANOVA uses out-of-sample cross-validation to provide a measure of the information contained in neural data about the variables of interest. These estimates can, in principle, also be used to decode experimental variables on individual trials. Due to this close relationship, and to highlight the link to the extensive multivariate decoding literature, we often refer to our results as decoding results.

### Cross-decoding

To achieve cross-condition decoding, we constructed contrast vectors C_train_ and C_test_ to only contain the conditions to be trained or tested on, respectively. We applied this to estimate neural information within and across the 2 task conditions (pre and post), as well as the 2 confidence levels, and the 2 choices. Additionally, we also used a model trained on all trials and tested it separately on correct and incorrect trials. To estimate whether information was shared between time points, we computed the pattern distinctness when using regression parameters B_train_ from one time point, and B_test_ from another. We repeated this for every pair of time points. In order to assess whether 2 variables shared a common representational space, we used cross-variable decoding. We implemented this by using a training contrast C_train_ differentiating between the levels of one variable, and a test contrast C_test_ differentiating between the levels of another. Before further processing, all decoding time courses were smoothed using a Hanning window (500 ms, full width at half maximum). Time–time generalization matrices were smoothed using a 2D, 100 ms Hanning window.

### Geometric visualization of representational similarity

We reconstructed low-dimensional geometric representations of neural activity in multiple conditions using the decoding results. Decoding and cross-decoding values between multiple variables define the distances and angles of condition difference vectors. We used these to plot subsets of conditions in 2D spaces defined by the axes spanned by 2 variables of interest. For example, in Fig 4G, the length of the choice and response vectors is given by the magnitude of choice and response information, respectively; the angle between both is given by the cross-decoding between the 2 variables. The mapping vector reflects the projection of mapping information onto the 2D space spanned by choice and response information, indicating that mapping is not represented as an interaction between choice and response.

### Searchlight analysis

We repeated our main analysis in a searchlight fashion, in order to estimate the spatiotemporal distribution of neural information throughout the trial. For each of the 457 sources, we used cvMANOVA on that source as well as its immediate neighbors, including all 3 dipole directions. In order to maintain comparability between sources, we normalized the resulting pattern distinctness values by the square root of the size of the searchlight [20,21]. After averaging over both hemispheres, we split the searchlight decoding results of all 457 sources into 4 distinct groups (occipital, temporal, central, frontal) based on a previous parcellation into 15 anatomical areas [68]. We then averaged within each of these areas to maximize the SNR of our MEG data with inherently low spatial resolution, in order to show the spatiotemporal dynamics of neural information. To quantify a shift in choice information from sensory to motor areas, we correlated, for each participant, the cortical distribution of choice information during each time point with the distribution of stimulus information during stimulus presentation (1.25 s to 3.25 s), and with the distribution of response information during response execution (from 5.5 s). Statistical significance was assessed using one-tailed cluster permutation tests.

### Expected cross-decoding

The maximal amount of shared information between 2 contexts depends on the amount of information available in each individual context. Thus, in order to assess whether 2 representations are different, the strength of both representations has to be taken into account and compared with the strength of the shared representation. We thus estimated the expected cross-decoding

$$E_{12}=\sqrt{\left|D_{1}D_{2}\right|}∙sign(D_{1})∙sign(D_{2})$$

where D_1_ and D_2_ denote the pattern distinctness in the 2 contexts. The cross-decoding D_12_ between both contexts would be expected to approach E_12_ for identical representations. Any cross-decoding values smaller than E_12_ indicate that the representations are not fully overlapping.

### Single-trial stimulus prediction

To test whether neural choice representations were informed by the stimulus, we projected each trial’s neural data onto the multivariate axis spanned by yes and no choices as defined by the cvMANOVA model. We then computed the sign of these single-trial estimates to assess whether it corresponded to the stimulus class.

### Eye movement control

While we ensured continuous fixation using an online eye movement control at the beginning of each trial, small eye movements can still plausibly confound MEG signals [69]. We thus repeated our main decoding analysis (Fig 2) using eye-tracking data. For this purpose, we selected the x-position, y-position, and pupil size signals and averaged them over both eyes. Additionally, we computed the eye position eccentricity as sqrt(x^2^+y^2^). We then applied the same decoding analysis using cvMANOVA, using these 4 channels. We split the 26 participants into the 13 with the highest and lowest choice information in their eye signals, respectively. This revealed that in a subset of participants, eye signals were predictive of choice. To test whether this could plausibly explain the neural choice information, we compared the choice decoding time courses in both splits. As neural choice decoding was, if anything, weaker in those participants with higher choice decoding from the eye signals, the neural decoding was unlikely to be explained by eye movements (S3 Fig).

### Statistical analysis

We assessed the statistical significance of information using cluster-based sign permutation tests. After determining temporally contiguous clusters during which pattern distinctness was higher than 0 (one-tailed *t* test over participants, *P* < 0.05), we randomly multiplied the information time-course of each participant 10,000 times with either 1 or −1. In each random permutation, we recomputed information clusters and determined the cluster mass of the strongest cluster. Each original cluster was assigned a *p*-value by comparing its size to the distribution of sizes of the random permutation’s strongest clusters. The same procedure was used for cross-decoding analyses, however, using two-tailed *t* tests as true cross-decoding can also be negative. We also tested differences in information using this strategy, namely, between response information during “yes” and “no” choices (Fig 4E). To test for differences between high- and low-confidence correct and error trials, we averaged data over appropriate time-periods (1.25 to 5.5 s for choice information) and used one-tailed *t* tests, as we had a clear unidirectional hypothesis derived from signal detection theory. To determine whether the multivariate patterns underlying 2 representations were significantly different, we tested whether the empirical cross-decoding was smaller than the expected cross-decoding, again using cluster-based sign permutation tests. Cross-temporal generalization and dynamics were assessed analogously, however, using 2D clusters.

### Software

All analyses were performed in MATLAB, using custom code as well as the Fieldtrip [70] and SPM toolboxes. For meta-d′ analyses, we used code from http://www.columbia.edu/~bsm2105/type2sdt/ [32].

## Supporting information

S1 Fig Task variables and analysis schematic. **(A)** Task conditions and behavioral responses. Each combination of the binary task variables constituted one of 32 separate conditions. Because behavioral variables were partially correlated among each other and with experimentally controlled variables (e.g., confidence vs. accuracy and choice vs. stimulus), the number of trials varied across the 32 unique conditions. The rows with variables below the histogram correspond to the contrast vectors employed in the cross-validated MANOVA. **(B)** We performed multivariate pattern analysis using cross-validated MANOVA to estimate the difference D between MEG source level patterns associated with the 2 levels of each task variable. Importantly, cross-validated MANOVA allowed to independently quantify information about each variable without confounding of other, potentially correlated variables. Each dot represents MEG activity during one of the 32 conditions. For example, choice information is computed as the contrast between all conditions containing “yes” trials and those containing “no” trials (middle), stimulus information as the contrast between conditions containing “signal” and “noise” trials (right). For our main analyses, this procedure was applied to the action-linked (“pre”) and action-independent (“post”) contexts separately. Using a cross-decoding framework, the angle between D_pre_ and D_post_ enabled us to assess the degree of similarity between representations of any given variable during action-linked and action-independent contexts.
(TIFF)

S2 Fig Motor cortical beta lateralization. Beta lateralization predictive of each trial’s response hand was computed for pre- and post-conditions in an individually localized source in motor cortex for each participant. Horizontal bars indicate significant clusters (cluster permutation, two-tailed, *P* < 0.05). Colored lines and shaded regions indicate the mean +/− SEM across participants.
(TIFF)

S3 Fig Choice information in eye-tracking data. **(A)** Choice information contained in eye traces (x and y position, eccentricity, and pupil size), split into the 13 participants with the highest decoding values, and the 13 participants with the lowest. Eye traces were thus predictive of choice in a subset of participants. **(B)** Choice information contained in MEG data, split into the same groups as in (A). Participants in whom the eye traces were predictive of choice did not show stronger decoding of choice from MEG data (one-tailed *t* test, *P* > 0.05 for all time points). This indicates that choice information in MEG was not driven by eye movements or pupil size. **(C)** There was no positive across-subject relationship between the amount of choice information contained in eye traces and the amount of choice information contained in MEG data. Colored lines and shaded regions indicate the mean +/− SEM of information across participants.
(TIFF)

S4 Fig Previous trial information. (A) There was no significant information about the previous choice, and information about the current choice remained significant when including previous choice as a variable. Horizontal lines indicate clusters of significant information (cluster permutation, *P* < 0.01, *N* = 23). (B) There was significant information about the previous motor response throughout the trial, but information about the current choice and response remained significant when including previous response as a variable. Horizontal lines indicate clusters of significant information (cluster permutation, *P* < 0.01, *N* = 25). Colored lines and shaded regions indicate the mean +/− SEM of information across participants.
(TIFF)

S5 Fig Stimulus and response representations do not exhibit properties of a decision variable. Time-averaged stimulus information (1.25 to 3.5 s) and response information (3.25 to 6.5 s) in correct and error, and high- and low-confidence trials. The model was trained on both correct and error trials, but trials were split by accuracy for testing. Stars denote significant differences (*P* < 0.05, two-tailed *t* tests, *N* = 19).
(TIFF)

S6 Fig Main results in a core subset of 19 participants. We replicated the main results of Figs 2 and 3, using data from only the same 19 participants that were used in the confidence analyses in Fig 5. (**A**) Neural information in the pre- (darker colors) and post-conditions (brighter colors), as well as empirical (grey) and expected cross-information. All conventions as in Fig 2. (**B**) Time-resolved stimulus (top), response (middle), and choice (bottom) information in 4 groups of sources, as in Fig 3. All conventions as in Fig 3. (**C**) Correlation of the cortical distribution of choice information with the distribution of peak stimulus information (red) and peak response information (yellow), as in Fig 3. All conventions as in Fig 3.
(TIFF)

S7 Fig Main results are similar between task versions. We replicated the main results of Fig 2, using data from each task version separately. (**A**) Neural information in the pre- (darker colors) and post-conditions (brighter colors), as well as empirical (grey) and expected cross-information in task version A. All conventions as in Fig 2. (**B**) Neural information in task version B.
(TIFF)

## References

[1] Cisek P, Kalaska JF. Neural Mechanisms for Interacting with a World Full of Action Choices. Annu Rev Neurosci. 2010;33:269–298. doi: 10.1146/annurev.neuro.051508.135409 20345247

[2] Shadlen MN, Newsome WT. Neural Basis of a Perceptual Decision in the Parietal Cortex (Area LIP) of the Rhesus Monkey. J Neurophysiol. 2001;86:1916–1936. doi: 10.1152/jn.2001.86.4.1916 11600651

[3] Gold JI, Shadlen MN. Representation of a perceptual decision in developing oculomotor commands. Nature. 2000;404:390–394. doi: 10.1038/35006062 10746726

[4] Gold JI, Shadlen MN. The Neural Basis of Decision Making. Annu Rev Neurosci. 2007;30:535–574. doi: 10.1146/annurev.neuro.29.051605.113038 17600525

[5] Wang M, Montanède C, Chandrasekaran C, Peixoto D, Shenoy KV, Kalaska JF. Macaque dorsal premotor cortex exhibits decision-related activity only when specific stimulus–response associations are known. Nat Commun. 2019:10. doi: 10.1038/s41467-019-09460-y 30996222 PMC6470163

[6] Gold JI, Shadlen MN. The Influence of Behavioral Context on the Representation of a Perceptual Decision in Developing Oculomotor Commands. J Neurosci. 2003;23:632–651. doi: 10.1523/JNEUROSCI.23-02-00632.2003 12533623 PMC6741872

[7] Nieder A, Wagener L, Rinnert P. A neural correlate of sensory consciousness in a corvid bird. Science. 2020;369:1626–1629. doi: 10.1126/science.abb1447 32973028

[8] Coallier É, Kalaska JF. Reach target selection in humans using ambiguous decision cues containing variable amounts of conflicting sensory evidence supporting each target choice. J Neurophysiol. 2014;112:2916–2938. doi: 10.1152/jn.00145.2014 25210160

[9] Bennur S, Gold JI. Distinct Representations of a Perceptual Decision and the Associated Oculomotor Plan in the Monkey Lateral Intraparietal Area. J Neurosci. 2011;31:913–921. doi: 10.1523/JNEUROSCI.4417-10.2011 21248116 PMC3380543

[10] Hebart MN, Donner TH, Haynes J-D. Human visual and parietal cortex encode visual choices independent of motor plans. NeuroImage. 2012;63:1393–1403. doi: 10.1016/j.neuroimage.2012.08.027 22922368

[11] Herding J, Spitzer B, Blankenburg F. Upper Beta Band Oscillations in Human Premotor Cortex Encode Subjective Choices in a Vibrotactile Comparison Task. J Cogn Neurosci. 2016;28:668–679. doi: 10.1162/jocn_a_00932 26836516

[12] Horwitz GD, Batista AP, Newsome WT. Representation of an Abstract Perceptual Decision in Macaque Superior Colliculus. J Neurophysiol. 2004;91:2281–2296. doi: 10.1152/jn.00872.2003 14711971

[13] Ludwig S, Herding J, Blankenburg F. Oscillatory EEG signatures of postponed somatosensory decisions. Hum Brain Mapp. 2018;39:3611–3624. doi: 10.1002/hbm.24198 29717524 PMC6866617

[14] Merten K, Nieder A. Comparison of abstract decision encoding in the monkey prefrontal cortex, the presupplementary, and cingulate motor areas. J Neurophysiol. 2013;110:19–32. doi: 10.1152/jn.00686.2012 23576697

[15] Merten K, Nieder A. Active encoding of decisions about stimulus absence in primate prefrontal cortex neurons. Proc Natl Acad Sci. 2012;109:6289–6294. doi: 10.1073/pnas.1121084109 22460793 PMC3341060

[16] Minxha J, Adolphs R, Fusi S, Mamelak AN, Rutishauser U. Flexible recruitment of memory-based choice representations by the human medial frontal cortex. Science. 2020;368:eaba3313. doi: 10.1126/science.aba3313 32586990 PMC7531893

[17] Zhou Y, Freedman DJ. Posterior parietal cortex plays a causal role in perceptual and categorical decisions. Science. 2019;6. 10.1126/science.aaw8347 PMC7346736 31296771

[18] Herding J, Ludwig S, Blankenburg F. Response-Modality-Specific Encoding of Human Choices in Upper Beta Band Oscillations during Vibrotactile Comparisons. Front Hum Neurosci. 2017:11. doi: 10.3389/fnhum.2017.00118 28360848 PMC5350154

[19] Wu Y, Velenosi LA, Schröder P, Ludwig S, Blankenburg F. Decoding vibrotactile choice independent of stimulus order and saccade selection during sequential comparisons. Hum Brain Mapp. 2019;40:1898–1907. doi: 10.1002/hbm.24499 30565343 PMC6865757

[20] Allefeld C, Haynes J-D. Searchlight-based multi-voxel pattern analysis of fMRI by cross-validated MANOVA. NeuroImage. 2014;89:345–357. doi: 10.1016/j.neuroimage.2013.11.043 24296330

[21] Christophel TB, Iamshchinina P, Yan C, Allefeld C, Haynes J-D. Cortical specialization for attended versus unattended working memory. Nat Neurosci. 2018;21:494–496. doi: 10.1038/s41593-018-0094-4 29507410

[22] Pape A-A, Siegel M. Motor cortex activity predicts response alternation during sensorimotor decisions. Nat Commun. 2016:7. doi: 10.1038/ncomms13098 27713396 PMC5059771

[23] Britten KH, Newsome WT, Shadlen MN, Celebrini S, Movshon JA. A relationship between behavioral choice and the visual responses of neurons in macaque MT. Vis Neurosci. 1996;13:87–100. doi: 10.1017/s095252380000715x 8730992

[24] Nienborg H, Cumming BG. Decision-related activity in sensory neurons reflects more than a neuron’s causal effect. Nature. 2009;459:89–92. doi: 10.1038/nature07821 19270683 PMC2917918

[25] Siegel M, Buschman TJ, Miller EK. Cortical information flow during flexible sensorimotor decisions. Science. 2015;348:1352–1355. doi: 10.1126/science.aab0551 26089513 PMC4721574

[26] Cisek P, Kalaska JF. Neural Correlates of Reaching Decisions in Dorsal Premotor Cortex: Specification of Multiple Direction Choices and Final Selection of Action. Neuron. 2005;45:801–814. doi: 10.1016/j.neuron.2005.01.027 15748854

[27] King J-R, Dehaene S. Characterizing the dynamics of mental representations: the temporal generalization method. Trends Cogn Sci. 2014;18:203–210. doi: 10.1016/j.tics.2014.01.002 24593982 PMC5635958

[28] Spaak E, Watanabe K, Funahashi S, Stokes MG. Stable and Dynamic Coding for Working Memory in Primate Prefrontal Cortex. J Neurosci. 2017;37:6503–6516. doi: 10.1523/JNEUROSCI.3364-16.2017 28559375 PMC5511881

[29] Zhao Y, Yates JL, Levi AJ, Huk AC, Park IM. Stimulus-choice (mis)alignment in primate area MT. Marinazzo D, editor. PLoS Comput Biol. 2020;16: e1007614. doi: 10.1371/journal.pcbi.1007614 32421716 PMC7259805

[30] Lueckmann J-M, Macke JH, Nienborg H. Can Serial Dependencies in Choices and Neural Activity Explain Choice Probabilities? J Neurosci. 2018;38:3495–3506. doi: 10.1523/JNEUROSCI.2225-17.2018 29440531 PMC5895039

[31] Urai AE, de Gee JW, Tsetsos K, Donner TH. Choice history biases subsequent evidence accumulation. elife. 2019:8. doi: 10.7554/eLife.46331 31264959 PMC6606080

[32] Maniscalco B, Lau H. A signal detection theoretic approach for estimating metacognitive sensitivity from confidence ratings. Conscious Cogn. 2012;21:422–430. doi: 10.1016/j.concog.2011.09.021 22071269

[33] Kiani R, Corthell L, Shadlen MN. Choice Certainty Is Informed by Both Evidence and Decision Time. Neuron. 2014;84:1329–1342. doi: 10.1016/j.neuron.2014.12.015 25521381 PMC4271191

[34] Grimaldi P, Lau H, Basso MA. There are things that we know that we know, and there are things that we do not know we do not know: Confidence in decision-making. Neurosci Biobehav Rev. 2015;55:88–97. doi: 10.1016/j.neubiorev.2015.04.006 25929444 PMC4501881

[35] Odegaard B, Grimaldi P, Cho SH, Peters MAK, Lau H, Basso MA. Superior colliculus neuronal ensemble activity signals optimal rather than subjective confidence. Proc Natl Acad Sci U S A. 2018;115:E1588–E1597. doi: 10.1073/pnas.1711628115 29382765 PMC5816145

[36] Maniscalco B, Odegaard B, Grimaldi P, Cho SH, Basso MA, Lau H, et al. Tuned inhibition in perceptual decision-making circuits can explain seemingly suboptimal confidence behavior. Beck J, editor. PLoS Comput Biol. 2021;17:e1008779. doi: 10.1371/journal.pcbi.1008779 33780449 PMC8032199

[37] Macmillan NA, Creelman CD. Detection Theory: A User’s Guide. Psychology Press; 2004.

[38] Hebart MN, Schriever Y, Donner TH, Haynes J-D. The Relationship between Perceptual Decision Variables and Confidence in the Human Brain. Cereb Cortex. 2016;26:118–130. doi: 10.1093/cercor/bhu181 25112281

[39] Shadlen MN, Kiani R. Decision Making as a Window on Cognition. Neuron. 2013;80:791–806. doi: 10.1016/j.neuron.2013.10.047 24183028 PMC3852636

[40] Kiani R, Shadlen MN. Representation of Confidence Associated with a Decision by Neurons in the Parietal Cortex. Science. 2009;324:759–764. doi: 10.1126/science.1169405 19423820 PMC2738936

[41] de Gee JW, Knapen T, Donner TH. Decision-related pupil dilation reflects upcoming choice and individual bias. Proc Natl Acad Sci U S A. 2014:111. doi: 10.1073/pnas.1317557111 24449874 PMC3918830

[42] Donner TH, Siegel M, Fries P, Engel AK. Buildup of Choice-Predictive Activity in Human Motor Cortex during Perceptual Decision Making. Curr Biol 2009;19:1581–1585. doi: 10.1016/j.cub.2009.07.066 19747828

[43] Tsetsos K, Pfeffer T, Jentgens P, Donner TH. Action Planning and the Timescale of Evidence Accumulation. Marshall JAR, editor. PLoS ONE. 2015;10:e0129473. doi: 10.1371/journal.pone.0129473 26068458 PMC4467085

[44] Okazawa G, Hatch CE, Mancoo A, Machens CK, Kiani R. Representational geometry of perceptual decisions in the monkey parietal cortex. Cell. 2021;184:3748–3761.e18. doi: 10.1016/j.cell.2021.05.022 34171308 PMC8273140

[45] Seideman JA, Stanford TR, Salinas E. A conflict between spatial selection and evidence accumulation in area LIP. Nat Commun. 2022;13:4463. doi: 10.1038/s41467-022-32209-z 35915096 PMC9343639

[46] O’Connell RG, Dockree PM, Kelly SP. A supramodal accumulation-to-bound signal that determines perceptual decisions in humans. Nat Neurosci. 2012;15:1729–1735. doi: 10.1038/nn.3248 23103963

[47] Twomey DM, Kelly SP, O’Connell RG. Abstract and Effector-Selective Decision Signals Exhibit Qualitatively Distinct Dynamics before Delayed Perceptual Reports. J Neurosci. 2016;36:7346–7352. doi: 10.1523/JNEUROSCI.4162-15.2016 27413146 PMC4945659

[48] Urai AE, Pfeffer T. An Action-Independent Signature of Perceptual Choice in the Human Brain. J Neurosci. 2014;34:5081–5082. doi: 10.1523/JNEUROSCI.0477-14.2014 24719086 PMC6609003

[49] Quinn KR, Seillier L, Butts DA, Nienborg H. Decision-related feedback in visual cortex lacks spatial selectivity. Nat Commun. 2021;12:4473. doi: 10.1038/s41467-021-24629-0 34294703 PMC8298450

[50] Wu Y, Velenosi LA, Blankenburg F. Response modality-dependent categorical choice representations for vibrotactile comparisons. NeuroImage. 2021;226:117592. doi: 10.1016/j.neuroimage.2020.117592 33248258

[51] Sandhaeger F, von Nicolai C, Miller EK, Siegel M. Monkey EEG links neuronal color and motion information across species and scales. Pasternak T, Gold JI, Wimmer K, Tootell R, editors. elife. 2019;8:e45645. doi: 10.7554/eLife.45645 31287792 PMC6615858

[52] Kriegeskorte N, Mur M, Ruff DA, Kiani R, Bodurka J, Esteky H, et al. Matching Categorical Object Representations in Inferior Temporal Cortex of Man and Monkey. Neuron. 2008;60:1126–1141. doi: 10.1016/j.neuron.2008.10.043 19109916 PMC3143574

[53] Cichy RM, Pantazis D, Oliva A. Resolving human object recognition in space and time. Nat Neurosci. 2014;17:455–462. doi: 10.1038/nn.3635 24464044 PMC4261693

[54] Wilming N, Murphy PR, Meyniel F, Donner TH. Large-scale dynamics of perceptual decision information across human cortex. Nat Commun. 2020:11. doi: 10.1038/s41467-020-18826-6 33037209 PMC7547662

[55] Li Hegner Y, Lindner A, Braun C. A somatosensory-to-motor cascade of cortical areas engaged in perceptual decision making during tactile pattern discrimination: Cortical Cascade During Tactile Decisions. Hum Brain Mapp. 2017;38:1172–1181. doi: 10.1002/hbm.23446 27767240 PMC6867017

[56] Klaes C, Westendorff S, Chakrabarti S, Gail A. Choosing Goals, Not Rules: Deciding among Rule-Based Action Plans. Neuron. 2011;70:536–548. doi: 10.1016/j.neuron.2011.02.053 21555078

[57] Pape A-A, Noury N, Siegel M. Motor actions influence subsequent sensorimotor decisions. Sci Rep. 2017:7. doi: 10.1038/s41598-017-16299-0 29162928 PMC5698410

[58] Cisek P. Making decisions through a distributed consensus. Curr Opin Neurobiol. 2012;22:927–936. doi: 10.1016/j.conb.2012.05.007 22683275

[59] Summerfield C, de Lange FP. Expectation in perceptual decision making: neural and computational mechanisms. Nat Rev Neurosci. 2014;15:745–756. doi: 10.1038/nrn3838 25315388

[60] Haefner RM, Berkes P, Fiser J. Perceptual Decision-Making as Probabilistic Inference by Neural Sampling. Neuron. 2016;90:649–660. doi: 10.1016/j.neuron.2016.03.020 27146267

[61] Padoa-Schioppa C. Neurobiology of Economic Choice: A Good-Based Model. Annu Rev Neurosci. 2011;34:333–359. doi: 10.1146/annurev-neuro-061010-113648 21456961 PMC3273993

[62] Freedman DJ, Assad JA. A proposed common neural mechanism for categorization and perceptual decisions. Nat Neurosci. 2011;14:143–146. doi: 10.1038/nn.2740 21270782

[63] van Driel J, Olivers CNL, Fahrenfort JJ. High-pass filtering artifacts in multivariate classification of neural time series data. J Neurosci Methods. 2021;352:109080. doi: 10.1016/j.jneumeth.2021.109080 33508412

[64] de Cheveigné A, Arzounian D. Robust detrending, rereferencing, outlier detection, and inpainting for multichannel data. NeuroImage. 2018;172:903–912. doi: 10.1016/j.neuroimage.2018.01.035 29448077 PMC5915520

[65] Nolte G. The magnetic lead field theorem in the quasi-static approximation and its use for magnetoencephalography forward calculation in realistic volume conductors. Phys Med Biol. 2003;48:3637–3652. doi: 10.1088/0031-9155/48/22/002 14680264

[66] Van Veen BD, van Drongelen W, Yuchtman M, Suzuki A. Localization of brain electrical activity via linearly constrained minimum variance spatial filtering. IEEE Trans Biomed Eng. 1997:44. doi: 10.1109/10.623056 9282479

[67] Hebart MN, Baker CI. Deconstructing multivariate decoding for the study of brain function. NeuroImage. 2018;180:4–18. doi: 10.1016/j.neuroimage.2017.08.005 28782682 PMC5797513

[68] Hipp JF, Siegel M. BOLD fMRI Correlation Reflects Frequency-Specific Neuronal Correlation. Curr Biol. 2015;25:1368–1374. doi: 10.1016/j.cub.2015.03.049 25936551

[69] Quax SC, Dijkstra N, van Staveren MJ, Bosch SE, van Gerven MAJ. Eye movements explain decodability during perception and cued attention in MEG. NeuroImage. 2019;195:444–453. doi: 10.1016/j.neuroimage.2019.03.069 30951848

[70] Oostenveld R, Fries P, Maris E, Schoffelen J-M. FieldTrip: Open Source Software for Advanced Analysis of MEG, EEG, and Invasive Electrophysiological Data. Comput Intell Neurosci. 2011;2011:1–9. doi: 10.1155/2011/156869 21253357 PMC3021840

