# Supplementary figures and images for: Abstract perceptual choice signals during action-linked decisions in the human brain
Source: PLoS Biol. 2023 Oct 10;21(10):e3002324. doi: 10.1371/journal.pbio.3002324 (PMC10564462; doi:10.1371/journal.pbio.3002324)

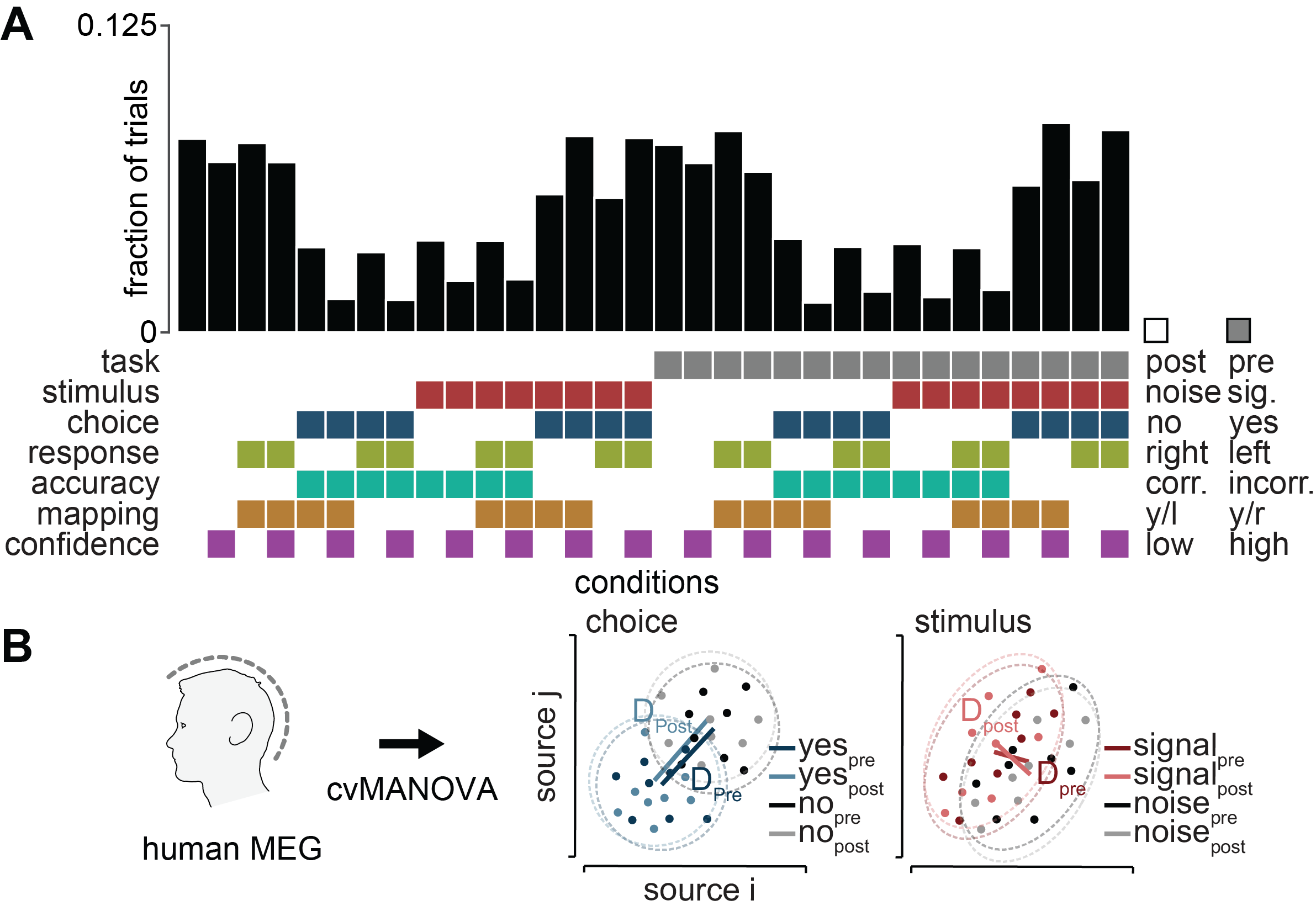

Supplement: S1 Fig — (A) Task conditions and behavioral responses. Each combination of the binary task variables constituted one of 32 separate conditions. Because behavioral variables were partially correlated among each other and with experimentally controlled variables (e.g., confidence vs. accuracy and choice vs. stimulus), the number of trials varied across the 32 unique conditions. The rows with variables below the histogram correspond to the contrast vectors employed in the cross-validated MANOVA. (B) We performed multivariate pattern analysis using cross-validated MANOVA to estimate the difference D between MEG source level patterns associated with the 2 levels of each task variable. Importantly, cross-validated MANOVA allowed to independently quantify information about each variable without confounding of other, potentially correlated variables. Each dot represents MEG activity during one of the 32 conditions. For example, choice information is computed as the contrast between all conditions containing “yes” trials and those containing “no” trials (middle), stimulus information as the contrast between conditions containing “signal” and “noise” trials (right). For our main analyses, this procedure was applied to the action-linked (“pre”) and action-independent (“post”) contexts separately. Using a cross-decoding framework, the angle between Dpre and Dpost enabled us to assess the degree of similarity between representations of any given variable during action-linked and action-independent contexts. (TIFF) [file pbio.3002324.s001.tiff]

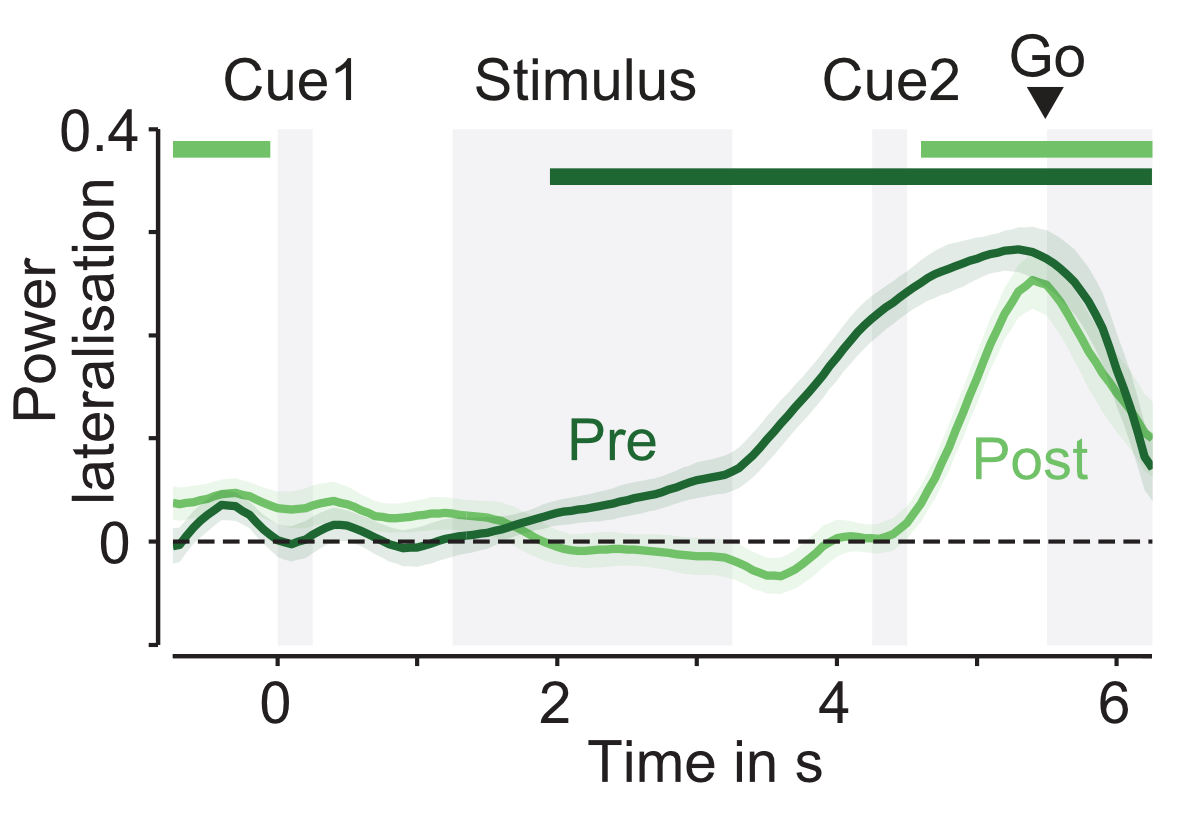

Supplement: S2 Fig — Beta lateralization predictive of each trial’s response hand was computed for pre- and post-conditions in an individually localized source in motor cortex for each participant. Horizontal bars indicate significant clusters (cluster permutation, two-tailed, P < 0.05). Colored lines and shaded regions indicate the mean +/− SEM across participants. (TIFF) [file pbio.3002324.s002.tiff]

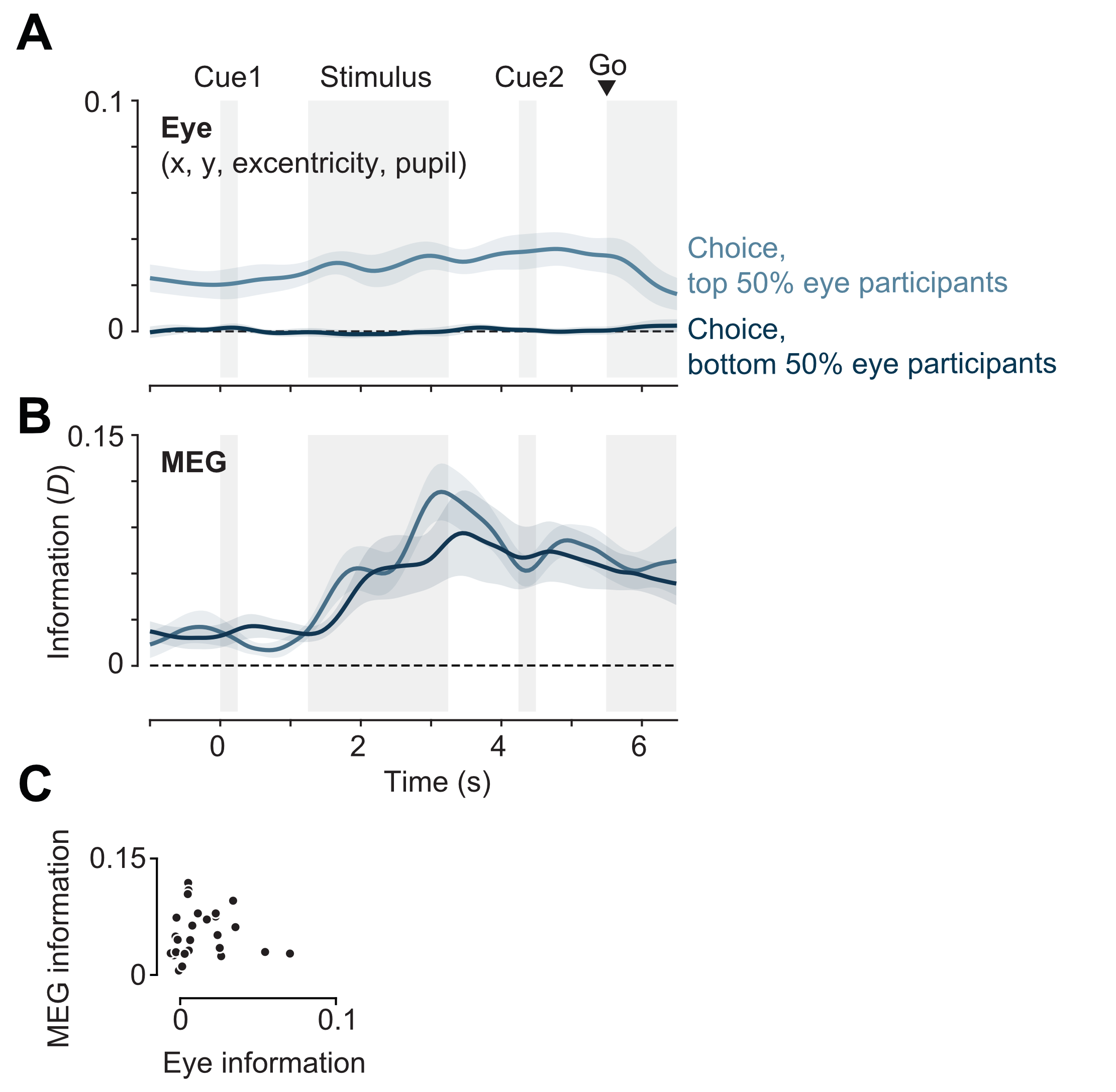

Supplement: S3 Fig — (A) Choice information contained in eye traces (x and y position, eccentricity, and pupil size), split into the 13 participants with the highest decoding values, and the 13 participants with the lowest. Eye traces were thus predictive of choice in a subset of participants. (B) Choice information contained in MEG data, split into the same groups as in (A). Participants in whom the eye traces were predictive of choice did not show stronger decoding of choice from MEG data (one-tailed t test, P > 0.05 for all time points). This indicates that choice information in MEG was not driven by eye movements or pupil size. (C) There was no positive across-subject relationship between the amount of choice information contained in eye traces and the amount of choice information contained in MEG data. Colored lines and shaded regions indicate the mean +/− SEM of information across participants. (TIFF) [file pbio.3002324.s003.tiff]

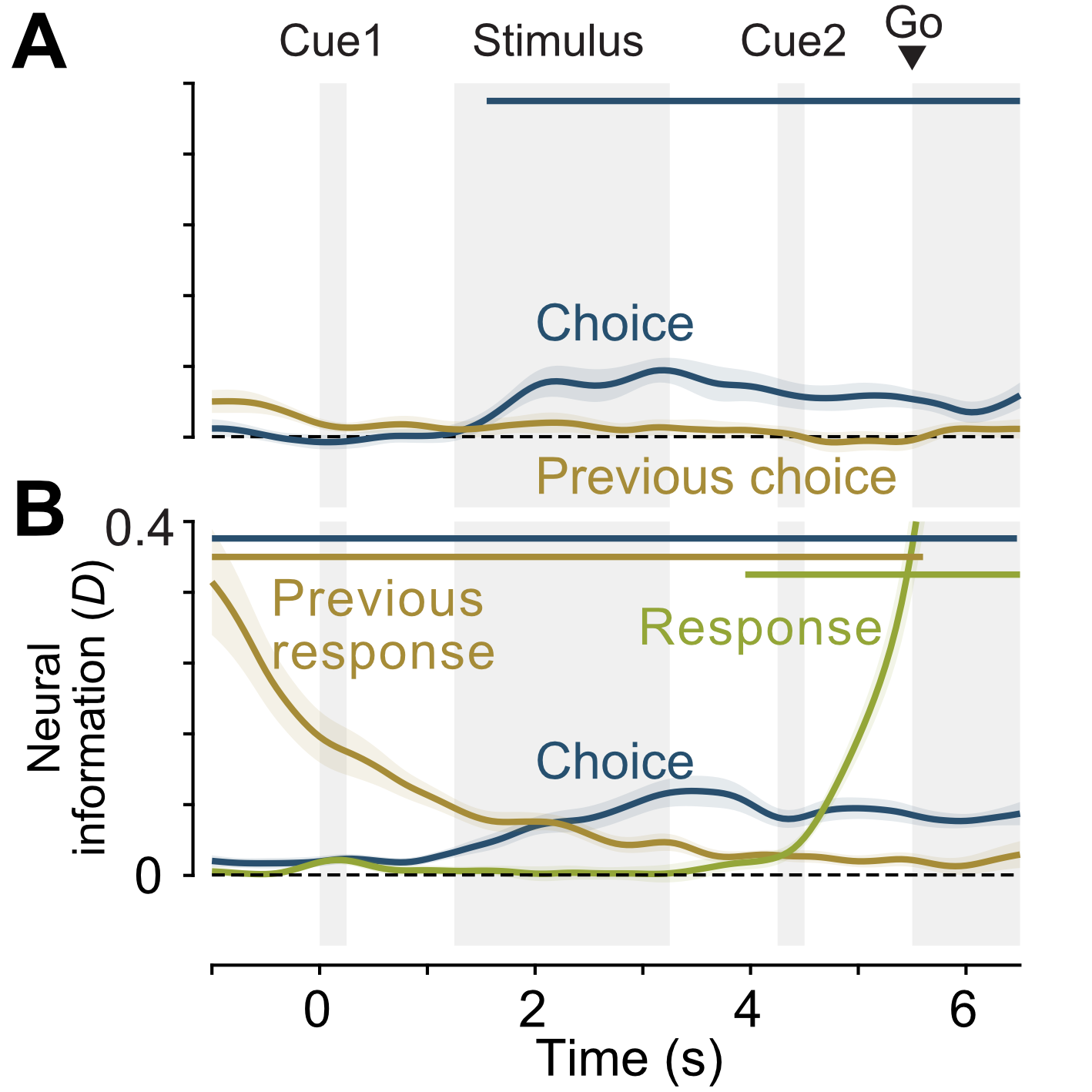

Supplement: S4 Fig — (A) There was no significant information about the previous choice, and information about the current choice remained significant when including previous choice as a variable. Horizontal lines indicate clusters of significant information (cluster permutation, P < 0.01, N = 23). (B) There was significant information about the previous motor response throughout the trial, but information about the current choice and response remained significant when including previous response as a variable. Horizontal lines indicate clusters of significant information (cluster permutation, P < 0.01, N = 25). Colored lines and shaded regions indicate the mean +/− SEM of information across participants. (TIFF) [file pbio.3002324.s004.tiff]

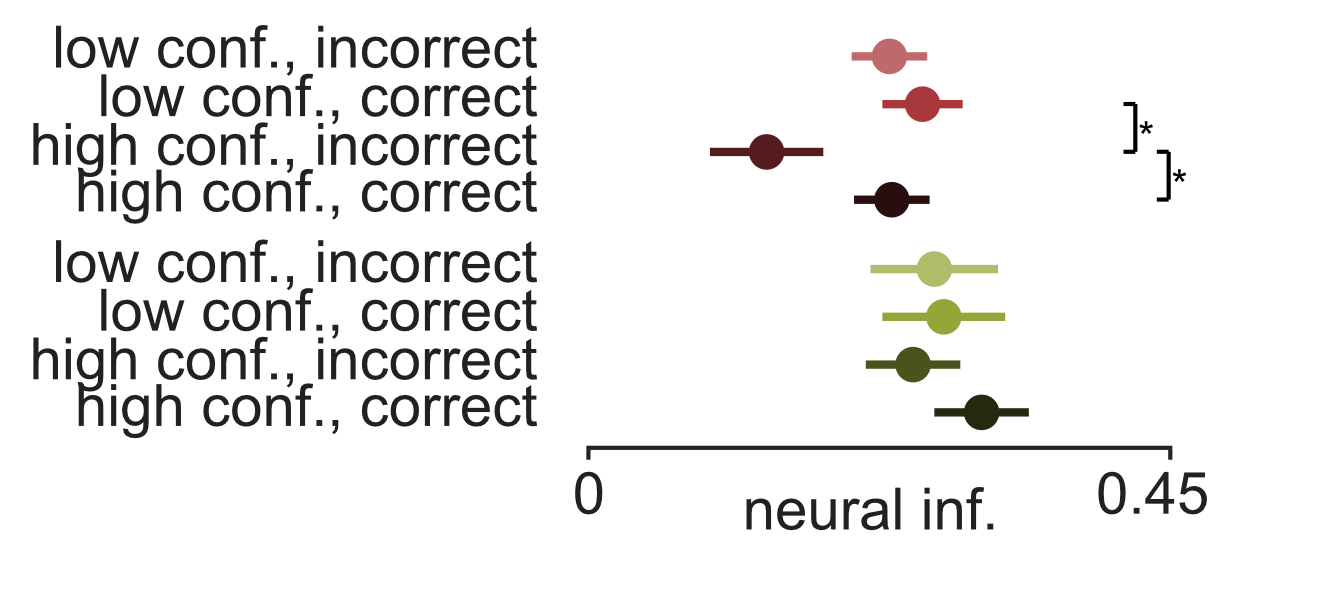

Supplement: S5 Fig — Time-averaged stimulus information (1.25 to 3.5 s) and response information (3.25 to 6.5 s) in correct and error, and high- and low-confidence trials. The model was trained on both correct and error trials, but trials were split by accuracy for testing. Stars denote significant differences (P < 0.05, two-tailed t tests, N = 19). (TIFF) [file pbio.3002324.s005.tiff]

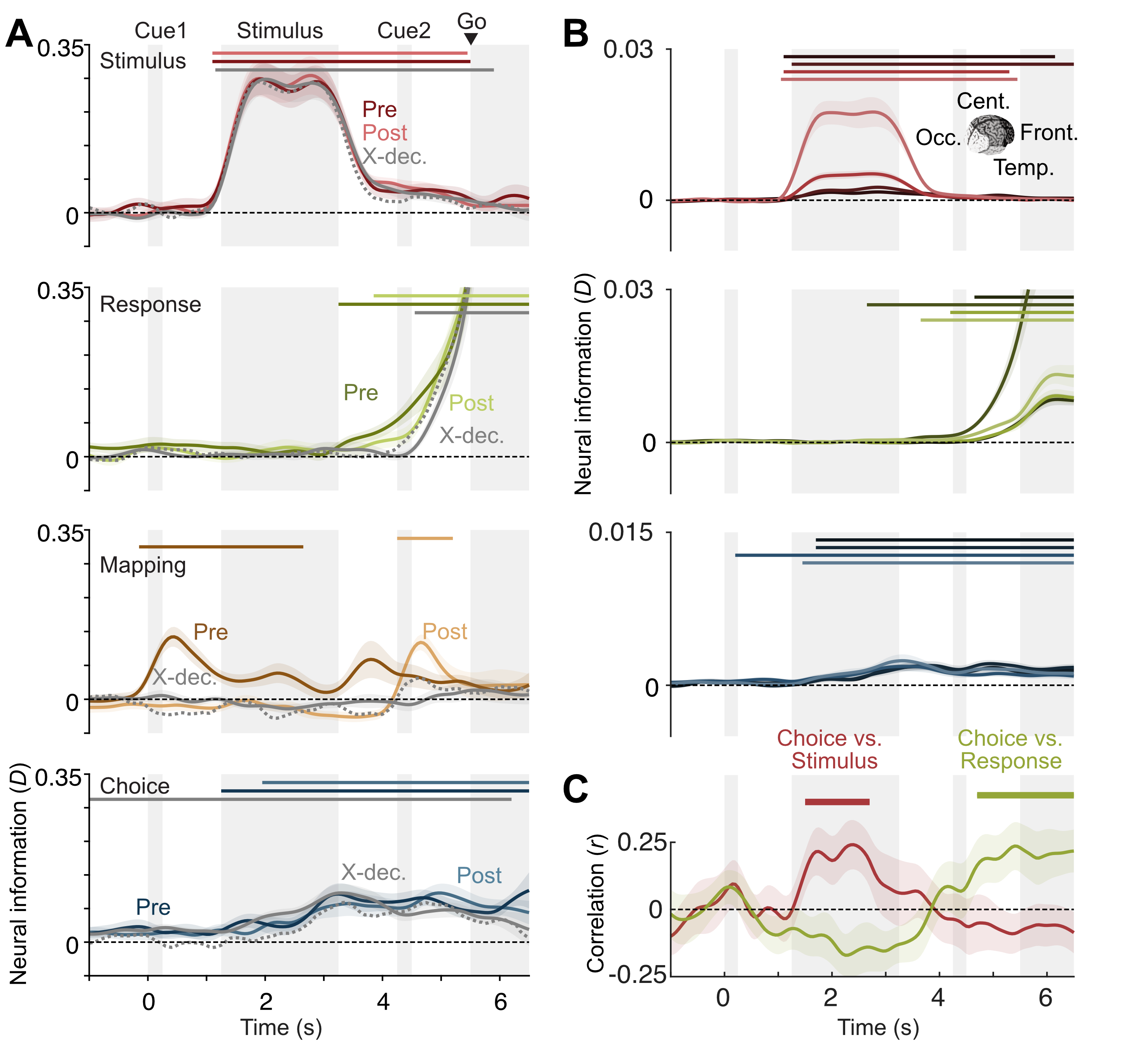

Supplement: S6 Fig — We replicated the main results of Figs 2 and 3, using data from only the same 19 participants that were used in the confidence analyses in Fig 5. (A) Neural information in the pre- (darker colors) and post-conditions (brighter colors), as well as empirical (grey) and expected cross-information. All conventions as in Fig 2. (B) Time-resolved stimulus (top), response (middle), and choice (bottom) information in 4 groups of sources, as in Fig 3. All conventions as in Fig 3. (C) Correlation of the cortical distribution of choice information with the distribution of peak stimulus information (red) and peak response information (yellow), as in Fig 3. All conventions as in Fig 3. (TIFF) [file pbio.3002324.s006.tiff]

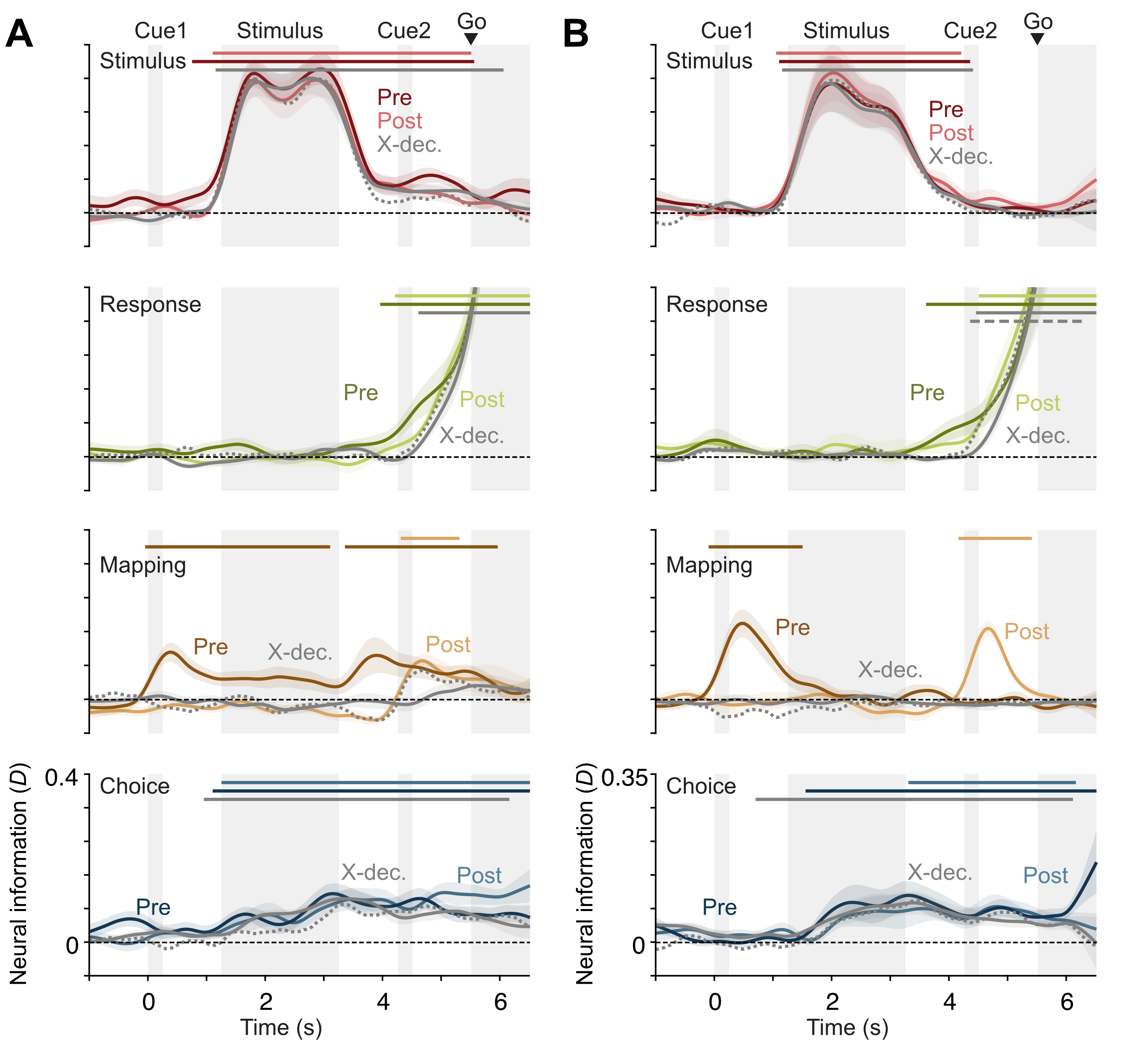

Supplement: S7 Fig — We replicated the main results of Fig 2, using data from each task version separately. (A) Neural information in the pre- (darker colors) and post-conditions (brighter colors), as well as empirical (grey) and expected cross-information in task version A. All conventions as in Fig 2. (B) Neural information in task version B. (TIFF) [file pbio.3002324.s007.tiff]
